# eIF3a Destabilization and TDP-43 Alter Dynamics of Heat-Induced Stress Granules

**DOI:** 10.3390/ijms22105164

**Published:** 2021-05-13

**Authors:** Ivana Malcova, Lenka Senohrabkova, Lenka Novakova, Jiri Hasek

**Affiliations:** 1 Institute of Microbiology, Czech Academy of Sciences, Videnska 1083, 14220 Prague, Czech Republic; L.Senohrabkova@seznam.cz (L.S.); lenkan@biomed.cas.cz (L.N.); hasek@biomed.cas.cz (J.H.); 2 First Faculty of Medicine, Charles University, Katerinska 42, 12108 Prague, Czech Republic

**Keywords:** Rpg1, eIF3, stress granules, heat shock, ER, mitochondria, ERMES, TDP-43, Hsp104, yeast

## Abstract

Stress granules (SGs) are membrane-less assemblies arising upon various stresses in eukaryotic cells. They sequester mRNAs and proteins from stressful conditions and modulate gene expression to enable cells to resume translation and growth after stress relief. SGs containing the translation initiation factor eIF3a/Rpg1 arise in yeast cells upon robust heat shock (HS) at 46 °C only. We demonstrate that the destabilization of Rpg1 within the PCI domain in the Rpg1-3 variant leads to SGs assembly already at moderate HS at 42 °C. These are bona fide SGs arising upon translation arrest containing mRNAs, which are components of the translation machinery, and associating with P-bodies. HS SGs associate with endoplasmatic reticulum and mitochondria and their contact sites ERMES. Although Rpg1-3-labeled SGs arise at a lower temperature, their disassembly is delayed after HS at 46 °C. Remarkably, the delayed disassembly of HS SGs after the robust HS is reversed by TDP-43, which is a human protein connected with amyotrophic lateral sclerosis. TDP-43 colocalizes with HS SGs in yeast cells and facilitates cell regrowth after the stress relief. Based on our results, we propose yeast HS SGs labeled by Rpg1 and its variants as a novel model system to study functions of TDP-43 in stress granules disassembly.

## 1. Introduction

The cell response to robust environmental stresses is accompanied by the formation of stress granules (SGs) as a defense to minimize stress-related damage and to modulate gene expression to promote cell survival. SGs are dynamic membrane-less ribonucleoprotein (RNP) assemblies arising upon translational arrest, containing mRNAs, components of the translation machinery, RNA-binding, and non-RNA-binding proteins [1,2,3]. The composition of SGs depends on the type and duration of the stress, but the translation initiation factor eIF3 is a hallmark of canonical SGs [3,4,5,6,7,8]. mRNAs present in SGs were thought to be in a silent, translationally repressed state. However, some mRNAs can undergo translation, even when localized in SGs induced by oxidative stress in HeLa cells [9]. SGs interchange mRNAs with P-bodies [10] that are often observed in the vicinity of SGs [11]. P-bodies are RNPs characterized by mRNAs and proteins linked to RNA metabolism [12,13]. In contrast to SGs, P-bodies are also present at physiological conditions [14] and never contain eIF3.

Assembly of SGs is a multistep process that is at least biphasic. First, translation initiation is arrested upon stress. Inhibition of translation can occur via eIF2α-dependent or independent pathways. Still, the disassembly of polysomes mediated either by mTOR inhibition, phosphorylation of eIF2α, or interference with the eIF4F complex appears to be a universal trigger of SGs formation [3,15]. Second, mRNAs liberated from polysomes, and their associated proteins, condensate into distinct cytoplasmic foci. The central player of SGs condensation in mammalian cells is the G3BP protein [16,17]. Ded1 could be assigned as a “super-aggregator” under elevated temperature in yeast cells [18,19]. SGs as mRNA–protein condensates consist of a stable core and a more dynamic shell [2]. The SGs core is enriched in translation initiation factors, RNA-binding proteins, proteins with predicted prion-like domains (PrLDs), non-RNA binding proteins, ATP-dependent proteins, nucleic acid-remodeling complexes, proteins without PrLDs, and proteins involved in neurodegenerative diseases [2,20]. Longer and poorly translated mRNAs are also targeted to the SGs core [21]. Formation of the SGs core is thought to be initiated by an increasing pool of untranslated mRNAs with bound proteins containing intrinsically disordered regions (IDR) and low complexity sequences (LCS). These undergo liquid–liquid phase separation (LLPS) based on weak, dynamic interactions between IDR and LCS. In time, maturation occurs, and a stable core arises due to increased concentrations of core components. Then, the SGs core recruits shell components [2,22]. Next to IDR and LCS, also RNA recognition motifs (RRMs) are involved in the phase separation of proteins carrying them [23,24].

The disassembly of SGs has not been in the center of attention, in contrast to SGs formation. Therefore, many questions stay unanswered and only now are being addressed [25]. SGs disassembly is believed to be also a multistep process when the shell dissipates first, followed by the clearance of cores. During recovery after the stress relief, mRNAs are returned to translation or degraded if aberrant. The restoration of translation is a prerequisite for the resumption of cell growth. Similarly, proteins are assisted by molecular chaperones to resolve or degrade [26,27,28]. Molecular chaperones, including heat shock proteins (Hsps), represent the first line of defense against the accumulation of nonfunctional proteins in cells. Several structurally unrelated chaperones exist in cells, such as Hsp40, Hsp60, Hsp70, Hsp90, Hsp100, and small Hsp families, forming cooperative pathways and networks. Chaperones are involved in many other processes next to the refolding of stress-denatured proteins, including de novo folding of nascent proteins, oligomeric assembly, protein trafficking, and assistance in proteolytic degradation [29]. Hsp70 chaperones, together with their co-chaperones from the Hsp40 family, are key players in maintaining the proteostasis implicated not only in protein folding but in protein degradation, too [30,31]. Chaperones help proteins engage the native conformation, the thermodynamically most stable state, at minimum free energy and prevent their aggregation by transiently shielding exposed hydrophobic amino acid residues [32]. If refolding is not successful, eukaryotic cells evolved an intricate system called spatial protein quality control (SQC) to protect cells against proteotoxic stress by sequestering protein aggregates and misfolded proteins into stress-specific inclusion bodies and deposition sites [33,34]. SQC and protein degradation by the ubiquitin–proteasome system or autophagy help maintain cellular proteostasis, which is crucial for proper cell functions and cell survival under stress and physiological conditions.

The reversibility of their formation is an intrinsic characteristic of genuine SGs. However, the accumulation of misfolded proteins after the stress can overload the protein quality machinery, leading to persistent or aberrant SGs. Persistent SGs and deregulated protein aggregation impair cell fitness and aging, contributing to human diseases, including diabetes, cancers, and neurodegenerative diseases [35,36]. Yeasts represent a model complementary to higher eukaryotes for studies of pathophysiology and therapeutic possibilities of neurodegenerative diseases [37,38,39]. Amyotrophic lateral sclerosis (ALS) is a fatal disease affecting upper and lower motor neurons in the brain and spinal cord. Yeast ALS models include cells producing three human proteins, SOD1, FUS1, and TDP-43, which are associated with ALS pathologies [40,41]. With the help of large-scale screens in yeasts, searches for therapeutic strategies to attenuate TDP-43-associated cytotoxicity were performed [42,43]. TDP-43 has been linked to the formation and regulation of mRNPs arising under stress conditions, namely stress granules [44,45,46]. Recent findings suggest that the occurrence of cytoplasmic TDP-43 inclusions is complex, and SGs may facilitate, but are not required for, the aggregation of TDP-43 in the cytoplasm [47,48,49].

SGs induced by robust heat shock (HS) at 46 °C [6,27] represent genuine yeast SGs characterized by the presence of the translation initiation factor eIF3. Yeast eIF3 is composed of five subunits eIF3a/Rpg1, eIF3b/Prt1, eIF3c/Nip1, eIF3i/Tif34, and eIF3g/Tif35 [50]. This core loosely associates with eIF3j/Hcr1 [51], which is implicated more in translation termination than initiation [52]. The formation of HS SGs requires energy and actively translating mRNAs but is independent of eIF2α phosphorylation [6]. HS SGs include mRNAs, proteins involved in translation, RNA-binding proteins, and non-RNA binding proteins, and they are decorated by various chaperones [6,8,18,27,53]. The disassembly of 46 °C-HS SGs precedes the restoration of translation and is mediated by chaperone-assisted refolding [20,24,27]. Autophagy has also been suggested to be implicated in SGs disassembly [26]. Nevertheless, it remains unknown how the assembly and disassembly of HS SGs are regulated and what signaling pathways are involved. TORC1 (target of rapamycin complex 1) is recruited to SGs under robust HS, which causes its inhibition [54]. Catalytic subunits of protein kinase A, Tpk2 and Tpk3, have also been identified in HS SGs [55]. Recently, the recruitment of Cdc28/Cdk1 to SGs by Whi8 has been reported with a function for Cdk1 in SGs disassembly [56].

Although yeast eIF3a/Rpg1 responds to robust HS at 46 °C only, some translation machinery components such as translation elongation factors (eEF3/Yef3 and eEF1B/Tef4) and termination factor eRF1/Sup45, SGs proteins Ngr1 and Pub1, accumulate already upon moderate HS at 42 °C along with the marker protein of P-bodies Dcp2. These mRNPs need actively translating mRNAs, arise independently of eIF2α phosphorylation, and serve as “seeds” for genuine SGs assembling under robust HS [8]. In compliance with the described accumulation of some translation machinery components at 42 °C [8,18], the heat-induced condensation of Ded1, promoting a translational switch, has recently been described [19]. Ded1 phase-separates at 42 °C along with housekeeping mRNAs that become repressed. However, stress response transcripts that do not condensate with Ded1 are translated, thus enabling a proper stress response.

In this paper, we report on the formation of bona fide SGs by an Rpg1 variant, Rpg1-3, upon translational arrest at the moderate HS of 42 °C. The Rpg1-3-labeled 42 °C-HS SGs contain components of the translation machinery except for ribosomal subunits. mRNAs are also present in Rpg1-3-decorated SGs that are closely associated with P-body marker proteins. eIF2α is absent from 42 °C-HS SGs; however, eIF2A/YGR054w is a novel constituent of yeast heat-induced SGs. HS SGs formed at 42 °C and 46 °C were found associated with ER and mitochondria and their encounter sites ERMES. The disassembly of 42 °C-HS SGs is Hsp104-dependent, similar to 46 °C SGs. SGs formed in *rpg1-3* cells at 46 °C display a delayed disassembly compared to *RPG1* cells. Notably, human TDP-43 colocalizes with 42 °C- and 46 °C-HS SGs in yeast cells and, surprisingly, facilitates their disassembly and cell growth resumption after the stress. To our knowledge, this is the first indication for the implication of TDP-43 in the disassembly of heat-induced SGs in yeasts and mammals. Therefore, we propose yeast HS SGs decorated by Rpg1 and its variants as a novel model for further studies of TDP-43 functions in the cell recovery after the stress.

## 2. Results

### 2.1. eIF3a/Rpg1 Variant Induces Accumulation of the eIF3 Complex in Foci at Moderate Heat Shock

**Figure 1:**
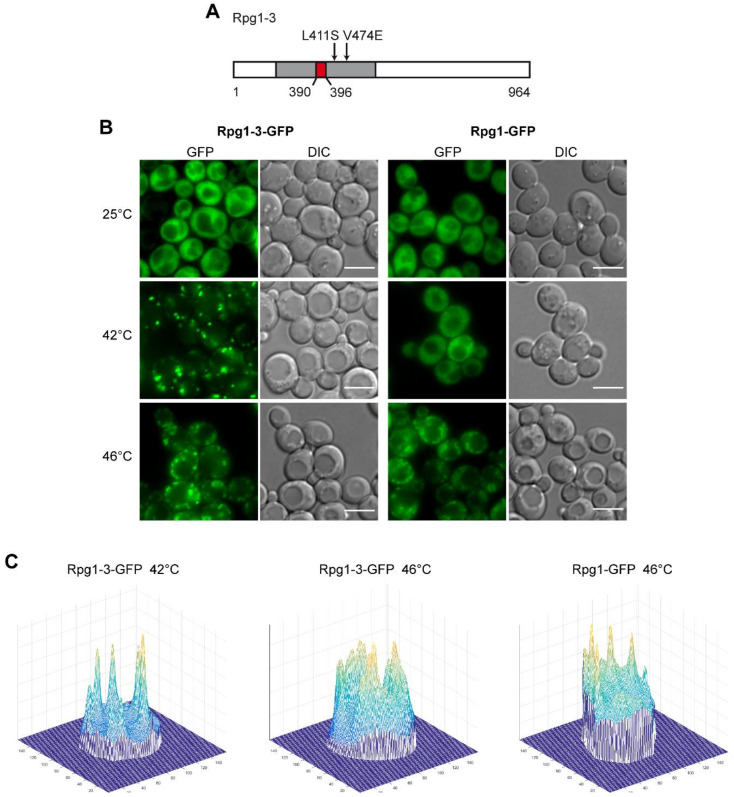
Cellular distribution of Rpg1 and Rpg1-3 upon heat stress. (**A**) A schematic presentation of the Rpg1-3 variant protein. Individual amino acid substitutions are depicted, and the deletion is indicated in red. The position of the PCI domain (amino acids 276–494, [59] is highlighted in gray). (**B**) Live-cell imaging of exponentially growing cells carrying Rpg1-3-GFP and Rpg1-GFP after HS for 30 min at 42 °C and 10 min at 46 °C. Scale bars, 5 µm. (**C**) Three-dimensional (3D)-brightness projections of typical cells carrying Rpg1-3-GFP after HS at 42 °C and 46 °C, and a representative cell of Rpg1-GFP after HS at 46 °C.

Our lab has established the translation initiation factor eIF3a/Rpg1 as the marker of heat shock-induced stress granules in yeast cells [6]. The Rpg1 protein, which is evenly distributed in the cytoplasm at physiological conditions, accumulates in SGs at a high temperature of 46 °C. In our previous work [57], we have described a thermosensitive variant of Rpg1, Rpg1-3, that carries a deletion of seven amino acid residues (390–396 AA) and two additional substitutions at L411S and V474E, which are all located within the PCI domain (Figure 1A). PCI (proteasome, COP9 signalosome, eIF3 complex) domains are present in proteins, forming multiprotein complexes. PCI domains are thought to mediate and stabilize protein–protein interactions within the complexes [58]. The Rpg1-3-GFP fusion protein was found to form reversible assemblies in around 15% of cells in a population grown at permissive conditions without any applied stress [57]. Here, we analyzed its behavior upon HS. The protein Rpg1-3 fused to GFP accumulated in foci after being exposed to 46 °C for 10 min, similarly to the Rpg1 protein. Notably, it also formed foci after a 30-min incubation at 42 °C in contrast to Rpg1 (Figure 1B).

There is a remarkable difference in the appearance of Rpg1-3-GFP foci after HS at 46 and 42 °C. Rpg1-3-GFP foci formed at 42 °C are less numerous, brighter, larger, and more condensed than those formed by the same protein at 46 °C. On the other hand, foci of Rpg1-3-GFP arising at 46 °C more resemble SGs of the Rpg1-GFP protein at 46 °C. The difference is well-documented on 3D-brightness projections made with a custom-written script oCellaris (see Section 4 for details) and presented in Figure 1C. The projections indicate that the level of diffused fluorescent signal in cells with Rpg1-3-GFP after HS 42 °C is lower than at 46 °C and in Rpg1-GFP producing cells upon HS 46 °C. It suggests that more of Rpg1-3-GFP is sequestered into foci after HS at 42 °C than 46 °C. Peaks visible on 3D-brightness projections correspond to individual foci and document that after HS at 42 °C, Rpg1-3-GFP foci are more defined and distinguishable from the remaining cytosolic signal than at 46 °C.

**Figure 2:**
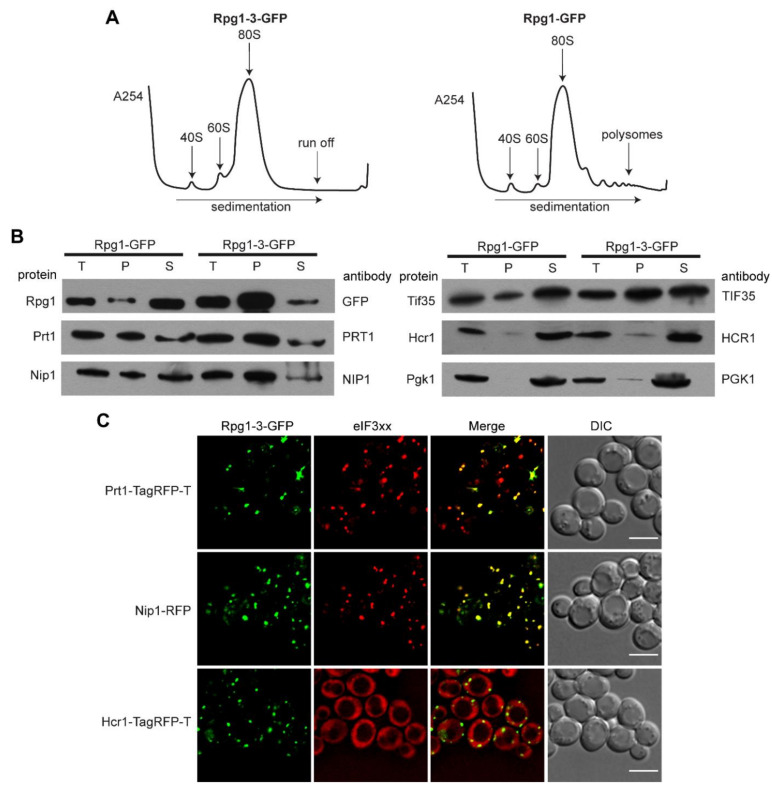
Rpg1-3-GFP foci formed at 42 °C also contain other subunits of the eIF3 complex. (**A**) Polysome profile analysis of exponentially growing cells carrying Rpg1-3-GFP and Rpg1-GFP upon heat shock 42 °C for 30 min. Arrows indicate positions of the 40S, 60S, monosome, and polysome. (**B**) Immunoblot of fractions from differential centrifugation of Rpg1-3-GFP and Rpg1-GFP producing cells after HS 42 °C for 30 min. Pgk1 was used as a non-aggregating control. (T)—total protein lysate before centrifugation, 2 % *v*/*v*; pellet (P) and supernatant (S) fractions, 20% *v*/*v* of the volume of input protein lysates. (**C**) Live-cell imaging of exponentially growing cells carrying Rpg1-3-GFP and another tagged eIF3 subunit, Prt1-TagRFP-T, Nip1-RFP, or Hcr1-TagRFP-T after HS 42 °C for 30 min. Single layers of Z-stacks deconvolved with the AMLE filter are shown. Scale bars, 5 µm.

Seeing the similarity of Rpg1-3-GFP foci at 46 °C with SGs formed by Rpg1-GFP upon robust HS at 46 °C, we analyzed polysome profiles to determine the status of translation in *rpg1-3* cells at 42 °C and 46 °C. The polysome profile analysis shown in Figure 2A demonstrates that cells harboring the *rpg1-3* allele as the only source of the essential Rpg1 protein completely arrest translation at 42 °C. Wild-type (WT) *RPG1* cells still displayed some polysomes after 30-min exposure to 42 °C. However, their level was significantly reduced compared to polysome profiles recorded in unstressed conditions and presented in Figure S1A. As expected, polysomes were not detected in both *rpg1-3* and *RPG1* cells after 10 min at 46 °C, which agrees with previously published results for the WT strain [6] (Figure S1A).

Next, we performed differential centrifugation to determine whether other subunits of the eIF3 complex accumulate in foci along with Rpg1-3 at HS 42 °C. Western blot analysis of supernatant and pellet fractions using respective antibodies (Figure 2B) showed that two eIF3a/Rpg1-interacting subunits eIF3b/Prt1 and eIF3c/Nip1 almost entirely followed Rpg1-3 into the pellet fraction. Another eIF3 subunit, eIF3g/Tif35, was found distributed in both the pellet and supernatant. However, eIF3j/Hcr1, the substoichiometric eIF3 component, was detected nearly entirely in the supernatant fraction. A weak signal of Hcr1 could be seen in the pellet fraction similarly to that of the non-aggregating control Pgk1 (Figure 2B).

To confirm the results of differential centrifugation, we performed live-cell imaging. Double-labeled strains carrying a plasmid-derived Rpg1-3-GFP fusion and one of the eIF3 subunits endogenously tagged at their C-terminus by mRFP or TagRFP-T were followed. Images presented in Figure 2C show that Prt1-TagRFP-T and Nip1-RFP fully colocalized with Rpg1-3-GFP after exposure to 42 °C for 30 min. In contrast, Hcr1-TagRFP-T stayed diffusely distributed in the cytoplasm at the same conditions. Similar results were obtained after exposing analyzed strains to robust HS for 10 min at 46 °C (Figure S1B) when Prt1-TagRFP-T and Nip1-RFP fully colocalized with Rpg1-3-GFP foci and Hcr1-TagRFP-T stayed diffused in the cytoplasm. Prt1-TagRFP-T and Nip1-RFP also colocalized with Rpg1-GFP upon HS for 10 min at 46 °C, while Hcr1-TagRFP-T did not change its diffused cytosolic distribution (Figure S1C).

A corresponding analysis of eIF3 subunits in the Rpg1-GFP producing strain after 30-min HS at 42 °C confirmed that the accumulation of Nip1-RFP at 42 °C depends on the behavior of the Rpg1-3-GFP protein, since Nip1-RFP stayed diffusely distributed in the cytoplasm of *RPG1-GFP* cells (Figure S2A). Images of Prt1-TagRFP-T distribution in the Rpg1-GFP producing strain indicate that Prt1 is destabilized by the C-terminal tagging, making it susceptible to moderate HS at 42 °C. Prt1-TagRFP-T seems to impose Rpg1-GFP to change its even cytosolic distribution and colocalize with Prt1-TagRFP-T in foci, as Prt1-TagRFP-T was detected in foci after the exposition of the single-labeled strain to 42 °C for 30 min (Figure S2B). This result suggests that even though the C-terminal tagging destabilized Prt1, tagged Prt1 was incorporated in the eIF3 complex.

Taken together, the presence of the Rpg1-3 variant in cells as the sole source of the essential Rpg1 protein leads to the translation arrest and accumulation of the translation initiation complex eIF3 in foci already upon exposition to moderate HS, which is in contrast to the WT Rpg1 protein that responds to robust HS at 46 °C only.

### 2.2. Rpg1-3 Foci Arising at Moderate Heat Stress Contain Translation Machinery Components but Not Ribosomal Subunits

The accumulation of translation initiation factors upon translation arrest is a hallmark of SGs formation. SGs composition varies according to stress, and some types of SGs include the 40S ribosomal subunit, translation initiation, elongation, and termination factors [3,5]. To get more insight into the composition of Rpg1-3 foci forming upon exposition to 42 °C, we followed the cellular distribution of several proteins involved in translation.

**Figure 3:**
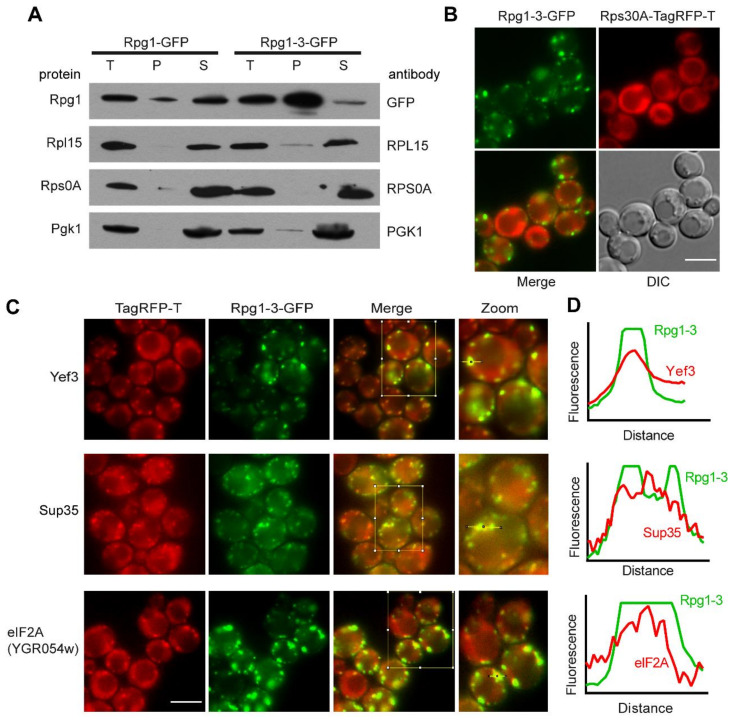
Rpg1-3 foci formed at 42 °C contain translation machinery components but not ribosomes. (**A**) Western blotting of samples from differential centrifugation of Rpg1-3-GFP producing cells after HS 42 °C 30 min. Pgk1 was included as a non-aggregating control. (T)—total protein lysate before centrifugation, 2% *v*/*v*; pellet (P) and supernatant (S) fractions, 20% *v*/*v* of the volume of input protein lysates. Exponentially growing cells carrying Rpg1-3-GFP were heat-shocked at 42 °C for 30 min, and colocalization of Rpg1-3-GFP foci with (**B**) Rps30-TagRFP-T (**C**) Yef3, Sup35, or eIF2A/YGR054w was followed by live-cell imaging. Scale bars, 5 µm. (**D**) Plot profiles of the fluorescence intensity along the yellow line in the zoomed images in (**C**).

First, we inspected pellet and supernatant fractions after differential centrifugation for the presence of small and large ribosomal subunits. We used antibodies against Rpl15, representing the 60S ribosomal subunit and Rps0A as a marker of 40S, respectively. The results shown in Figure 3A indicate that Rpg1-3 foci forming at 42 °C do not contain marker proteins of the 40S and 60S ribosomal subunits. Live-cell imaging brought the same answer regarding the 40S subunit, since no accumulation of Rps30A-TagRFP-T along with Rpg1-3-GFP could be observed after HS at 42 °C (Figure 3B).

Elongation factor eEF3/Yef3 has been identified as a component of 46 °C-HS SGs [8], and a spatial landmark of mRNPs formed at 42 °C on which other elements of SGs coalesce during robust HS at 46 °C [8].

Therefore, we inspected foci formed by Rpg1-3 at 42 °C for the presence of Yef3 in a double-labeled strain producing Rpg1-3-GFP and the Yef3 protein as an endogenous C-terminal TagRFP-T fusion. Yef3 responds to 42 °C differently in cells carrying Rpg1-3-GFP and Rpg1-GFP. In the *RPG1* strain, faint foci of Yef3 could be detected (Figure S3A), which corresponds to the published observation [8]. However, in *rpg1-3* cells, Yef3 foci were much brighter and more condensed and followed the distribution of Rpg1-3-GFP after 30 min at 42 °C, as demonstrated in Figure 3C. A clear colocalization of both fluorescence signals was confirmed by the fluorescence intensity profiles (Figure 3D).

The translation termination factor eRF3/Sup35, a component of 46 °C-HS SGs, has been previously reported to stay diffusely localized in the cytoplasm at 42 °C [6,8]. It should be noted that it was impossible to tag Sup35 endogenously at its C-terminus in the strain with already tagged Rpg1 protein. A diploid strain *SUP35/SUP35-TagRFP-T* carrying one intact copy of the *SUP35* gene had to be prepared by crossing *SUP35-TagRFP-T* haploid strain with either *rpg1-3* or *RPG1* strain. Live-cell imaging performed with the resulting diploid strain showed the Sup35-TagRFP-T fusion protein colocalized with Rpg1-3-GFP in foci after HS 42 °C (Figure 3C). Plots of intensity profiles of both fluorescence signals confirmed this observation (Figure 3D).

The translation initiation factor eIF2α/Sui2, which is responsible for the delivery of Met-tRNA_i_ to the 40 S subunit, did not form visible foci that could be assigned as colocalizing or associating with Rpg1-3 foci formed after exposition to 42 °C (data not shown). eIF2α was also absent from SGs formed by Rpg1 upon robust HS at 46 °C [8]. The protein encoded by the *SUI2* gene is the only eIF2α in yeast cells. However, yeast possesses an orthologue of the mammalian translation initiation factor eIF2A encoded by the open reading frame YGR054w. Mammalian eIF2A is thought to bind Met-tRNA_i_ to 40S independently of GTP but dependent on the AUG codon. Yeast eIF2A/YGR054w has been found to function as a suppressor of *URE2* internal ribosome entry site (IRES)-mediated translation in yeast cells [60]. We have tagged eIF2A/YGR054w endogenously with TagRFP-T in *rpg1-3* and *RPG1* cells and followed its cellular distribution by live-cell imaging. eIF2A accumulated in foci in *rpg1-3* cells after exposition to 42 °C. These foci overlapped with Rpg1-3-GFP, although eIF2A-TagRFP-T foci were not as robust and bright as Rpg1-3 foci and resembled more the foci of Yef3-TagRFP-T described above. In *RPG1* cells, the Rpg1-GFP signal stayed diffused in the cytoplasm, and foci of eIF2A-TagRFP-T were barely detectable (Figure S3A). It should be noted that the level of eIF2A-TagRFP-T fluorescence in cells of all observed strains was shallow, suggesting a low abundance of the protein within yeast cells. Upon HS at 46 °C, eIF2A-TagRFP-T colocalized with the Rpg1-3-GFP foci and SGs formed by Rpg1-GFP (Figure S3B).

Our results demonstrate that Rpg1-3-GFP foci formed upon moderate heat shock at 42 °C are similar to stress granules labeled by Rpg1-GFP during robust heat shock at 46 °C. They include translation elongation factor Yef3, translation termination factor Sup35, and translation initiation factor eIF2A/YGR054w but neither small nor large ribosomal subunits.

### 2.3. Rpg1-3 Foci Formed at 42 °C Include mRNAs and Associate with P-Bodies

**Figure 4:**
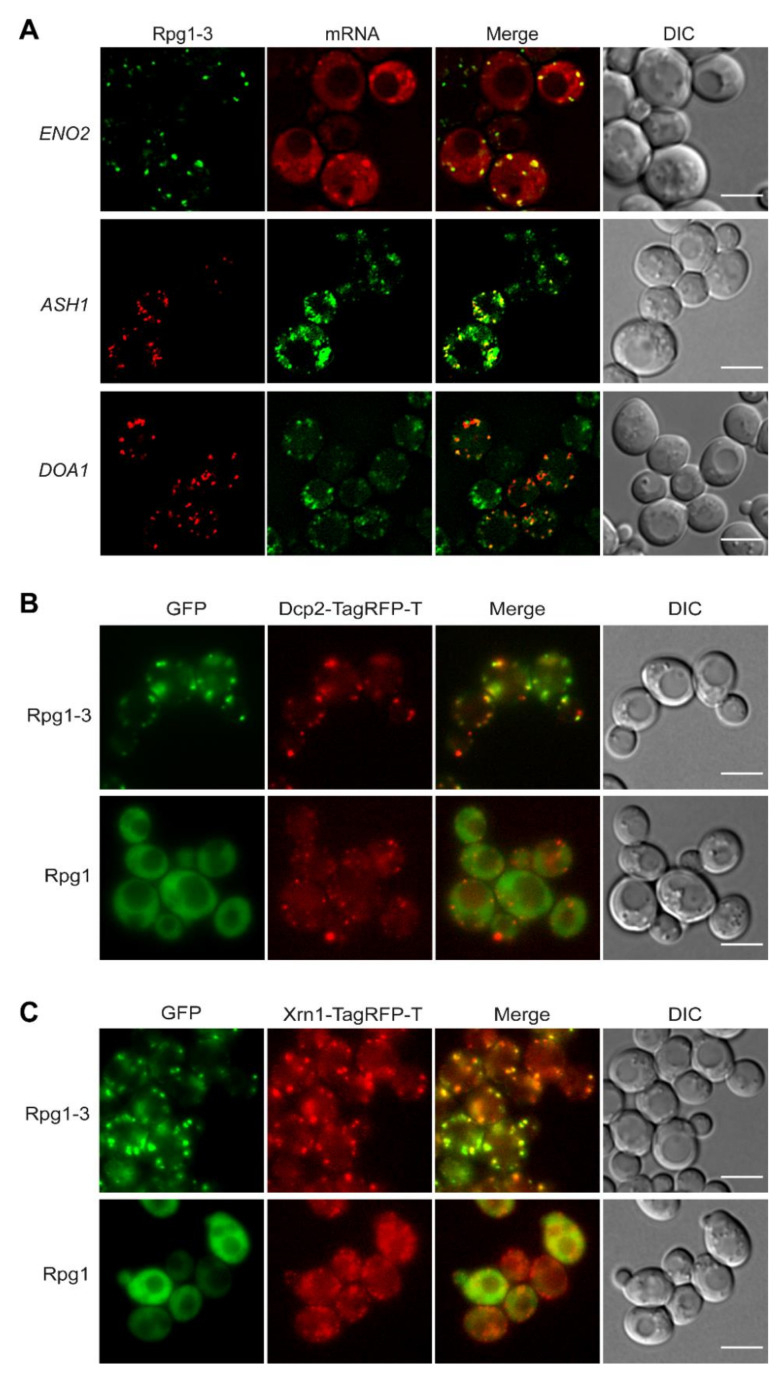
Distribution of mRNAs and P-bodies relative to Rpg1-3. (**A**) Live-cell imaging of exponentially growing cells heat-shocked at 42 °C for 30 min and carrying either Rpg1-3-GFP with *ENO2*-mCherry, Rpg1-3-TagRFP-T with *ASH1*-GFP, and Rpg1-3-TagRFP-T with *DOA1*-GFP. Single layers of Z-stacks after deconvolution with the AMLE filter (Xcellence software, Olympus) are presented. Association of Rpg1-3 foci with P-body marker proteins (**B**) Dcp2-TagRFP-T or (**C**) Xrn1-TagRFP-T after HS for 30 min at 42 °C. Single layers of Z-stacks are shown. Scale bars, 5 µm.

Stress granules are generally regarded as mRNPs since they always contain mRNAs and RNA-binding proteins. Even more, mRNAs and RNA-binding proteins are at the beginning of the formation of SGs [3]. To elucidate the presence of mRNAs in Rpg1-3 foci at 42 °C, we employed strains in which mRNAs were labeled either with the original MS2-CP [61] or a re-engineered MS2-MCP [62] systems. First, we used a strain carrying rpg1-3-GFP as the sole copy of the RPG1 gene and MS2-CP-labeled ENO2 mRNA and analyzed it under HS at 42 °C for 30 min. As shown in Figure 4A, Rpg1-3-GFP formed foci of typical appearance colocalizing with foci of ENO2-mCherry mRNA. The same result was obtained after HS for 10 min at 46 °C (Figure S4A). Enolase II encoded by the ENO2 gene is a metabolic enzyme involved in glycolysis; therefore, it is well visible in cells exponentially growing on glucose. To monitor other types of mRNA, we have chosen the ASH1 mRNA as an example of a regulated mRNA transported to the site of its translation in daughter cell [63] and constitutive DOA1 mRNA that might represent an organelle (mitochondria)-associated mRNA. Strains for the visualization of these mRNAs carry both mRNAs tagged with the MS2-MCP system and were obtained from R. Singer [62]. We introduced Rpg1-3 as a C-terminal fusion with TagRFP-T on a pIM23 vector [64] and observed the cells after HS at 42 °C for 30 min. Live-cell imaging revealed that Rpg1-3-TagRFP-T foci were associated with foci formed by GFP-labeled ASH1 mRNA in mother and daughter cells, although not all foci of ASH1 mRNA were decorated by Rpg1-3-TagRFP-T (Figure 4A). In contrast, foci of GFP-labeled DOA1 mRNA displayed a distribution similar to Rpg1-3-TagRFP-T foci and clearly associated with them (Figure 4A).

Another type of RNPs arising upon stress but existing also under physiological conditions is P-body [65]. P-body proteins colocalized with SGs formed by Rpg1 upon robust HS 46 °C [6]. Therefore, we inspected the cellular distribution of P-body markers Dcp2 and Xrn1 in relation to that of the Rpg1-3 protein. We used double-labeled strains carrying Rpg1-3-GFP and one of the P-body proteins as an endogenous C-terminal TagRFP-T fusion. First, we monitored the distribution of Rpg1-3 foci relative to Dcp2. In unstressed conditions (Figure S4B), Dcp2 is present in *rpg1-3* cells as tiny or, in some cells, larger foci. Upon HS for 30 min at 42 °C, Rpg1-3-GFP foci associate but do not overlap with Dcp2-TagRFP-T in larger foci (Figure 4B). Video S1 (in the Supplements) nicely documents the association and shows adjacent foci of Rpg1-3 and Dcp2 moving together during recovery from the stress. The Dcp2-TagRFP-T protein in 42 °C-exposed *RPG1* cells (Figure 4A) did not condensate into larger foci, and its distribution more resembled that observed in unstressed *rpg1-3* cells (Figure S4B).

The association of Rpg1-3 foci with P-bodies is further supported by images showing the different distribution of the exoribonuclease Xrn1 in *rpg1-3* cells compared to *RPG1* cells upon HS at 42 °C (Figure 4C). Xrn1-TagRFP-T produced in *RPG1* cells is present mainly in small foci at 42 °C, unlike in *rpg1-3* cells, where it forms large and dense foci associated with Rpg1-3-GFP foci. Thus, it is clear that the translational arrest in *rpg1-3* cells at 42 °C induces SGs and also promotes condensation of P-bodies.

Taken together, our results confirmed that Rpg1-3 foci formed upon moderate HS at 42 °C are mRNPs, since they include mRNAs. Given this and the presence of various translation machinery components, these Rpg1-3 foci can be regarded as stress granules missing ribosomal subunits. Therefore, we will refer to them as SGs hereafter. HS SGs labeled by Rpg1-3 at 42 °C, similarly to SGs decorated by Rpg1 at 46 °C, tightly associate with P-bodies.

### 2.4. Heat Shock-Induced SGs Associate with Endoplasmic Reticulum and Mitochondria, and Their Contact Sites ERMES

Given the above results on the association of HS SGs induced at 42 °C and decorated by Rpg1-3 with the mRNA of the protein linked by function to mitochondria, and our previous findings on the association of Rpg1-3 foci arising at physiological conditions [57] with the perinuclear and cortical endoplasmic reticulum (ER) and with mitochondria, we next followed the distribution of heat shock-induced SGs in yeast cells in relation to both organelles.

**Figure 5:**
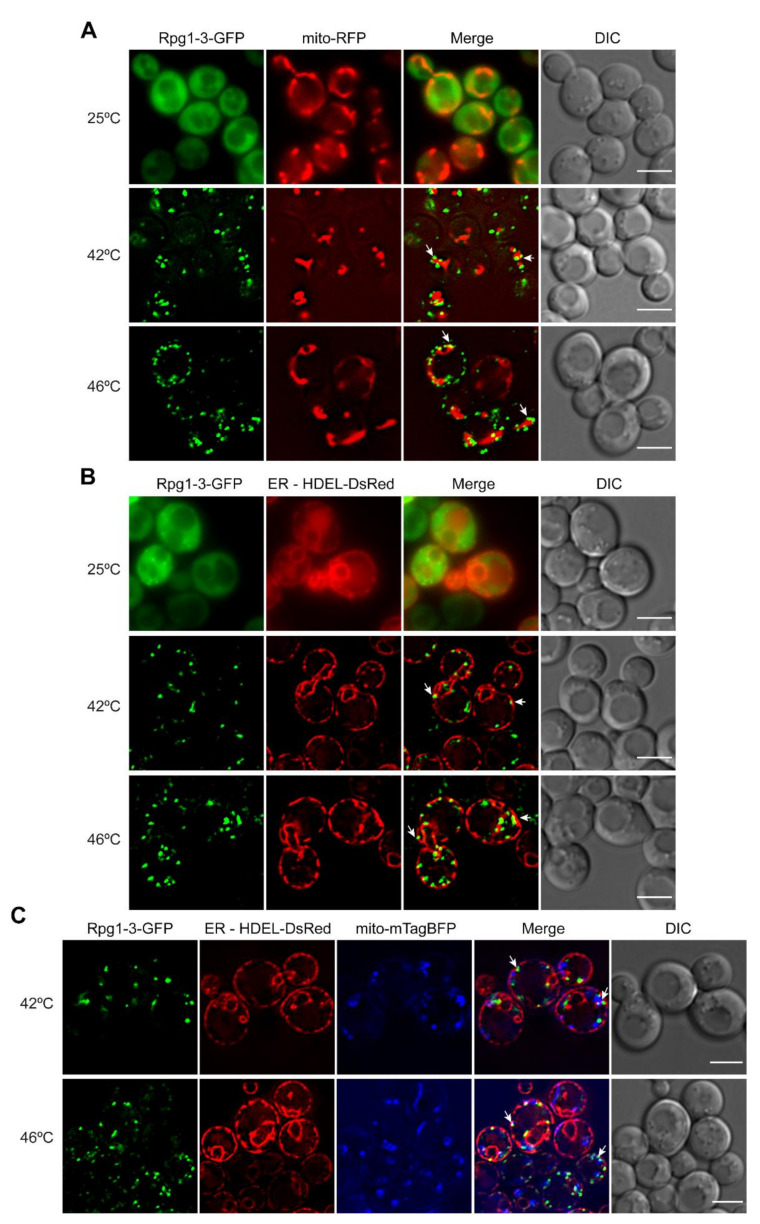
Association of HS SGs with mitochondria and ER. Live-cell imaging of exponentially growing cells at 25 °C and heat-shocked at 42 °C for 30 min or 46 °C for 10 min carrying Rpg1-3-GFP with (**A**) mito-RFP (mitochondrial marker) or (**B**) HDEL-DsRed (ER marker). (**C**) Distribution of Rpg1-3-GFP-decorated SGs at both temperatures in cells with simultaneously labeled mitochondria (mito-mTagBFP) and ER (HDEL-DsRed). White arrows point to HS SGs associating with organelles. Single layers of Z-stacks are presented for unstressed cells, and single layers of Z-stacks after deconvolution with the AMLE filter (Xcellence software, Olympus) are shown as HS images. Scale bars, 5 µm.

We introduced a mitochondrial targeting sequence Su9 in a fusion with RFP on the pYX142 plasmid (mito-RFP) to label mitochondria in *rpg1*-3 and *RPG1* cells and subjected them to moderate and robust heat shock. Live-cell imaging of unstressed cells showed a normal distribution of mitochondria and Rpg1-3-GFP diffusely localized in most cells. After the heat shock, either at 42 °C or 46 °C, Rpg1-3-GFP formed SGs often associated with collapsed mitochondria (Figure 5A). The association of Rpg1-GFP-labeled SGs at 46 °C with mitochondria was also detected but was not as evident as for Rpg1-3-GFP (Figure S5A).

Next, we introduced an ER marker HDEL-DsRed into *rpg1-3* and *RPG1* cells and followed the distribution of both fluorescent protein fusions under physiological and HS conditions relative to ER. In some unstressed *rpg1-3* cells, the foci of Rpg1-3-GFP associating with ER could be seen (Figure 5B). In contrast to mitochondria, neither perinuclear nor cortical ER changed their structure remarkably upon HS conditions. Rpg1-3-GFP decorated SGs observed in stressed cells associated with the perinuclear and cortical ER in every cell at both HS conditions (Figure 5B). The cortical ER depicts the cell periphery; therefore, it is well visible that Rpg1-3-labeled SGs induced at both HS conditions are located at the cell periphery mostly. After HS at 46 °C, SGs of Rpg1-GFP were similarly distributed at the cell periphery, and their association with the cortical and perinuclear ER was evident (Figure S5B).

The distribution of HS SGs in relation to both organelles was followed in a triple-labeled strain producing HDEL-DsRed, mito-mTagBFP, and Rpg1-3-GFP in one cell. Images recorded with triple-labeled cells upon both conditions of HS, 42 °C and 46 °C, confirmed the localization of SGs labeled with the Rpg1-3-GFP fusion protein in the vicinity to both mitochondria and ER (Figure 5C).

**Figure 6:**
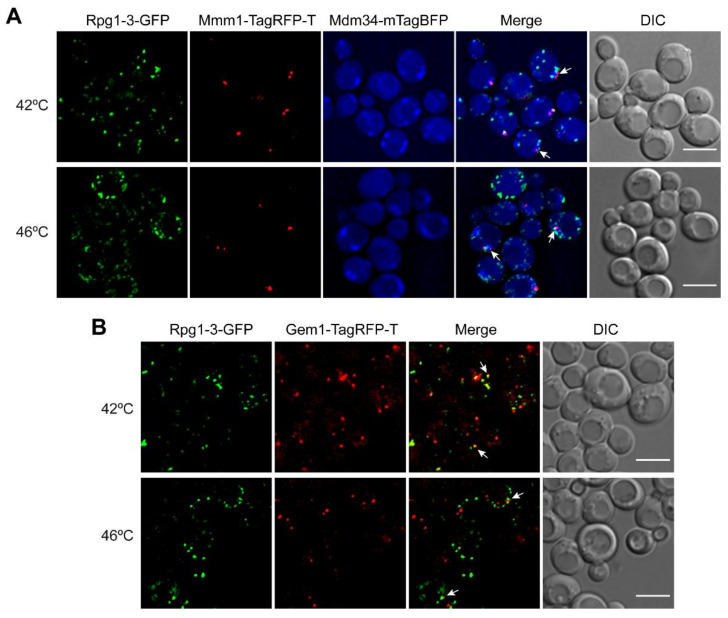
Heat stress-induced SGs in yeast cells associate with ERMES connecting ER and mitochondria. Live-cell imaging of exponentially growing cells heat-shocked at 42 °C for 30 min or 46 °C for 10 min carrying Rpg1-3-GFP with (**A**) ERMES components Mmm1-TagRFP-T and Mdm34-mTagBFP or (**B**) ERMES regulatory protein Gem1-TagRFP-T. White arrows point to SGs associating with ERMES. Single layers of Z-stacks after deconvolution with the AMLE filter (Xcellence software, Olympus) are shown. Scale bars, 5 µm.

Rpg1-3-labeled HS SGs were often situated at a site where both ER and mitochondria were close. The contact site between these two organelles is called ERMES for the ER–mitochondria encounter structure [66]. ERMES functions as a molecular bridge between ER and mitochondria, facilitating inter-organelle calcium and phospholipid exchange [66]. ERMES sites are present in a low number, up to 10 per cell. We hypothesized that HS SGs might associate with ERMES, particularly HS SGs at 42 °C that are less numerous than SGs at 46 °C and correspond to ERMES frequency in cells. We constructed a new strain on the background of Rpg1-3-GFP with tagged ER and mitochondrial ERMES components to support our hypothesis. Mmm1, an ER-membrane integral protein, was endogenously tagged with TagRFP-T at its C-terminus. Similarly, we labeled Mdm34, a mitochondrial outer membrane protein, with mTagBFP. The strain was subjected to HS at either 42 °C or 46 °C, and acquired images are displayed in Figure 6A. Indeed, we could detect 42 °C-HS SGs of Rpg1-3 in close contact with both ERMES components. Rpg1-3-GFP-labeled SGs at 46 °C also associated with the ERMES proteins; however, this was to a lesser extent, since they are more numerous than 42 °C-HS SGs and ERMES.

To bring another proof of the association of HS SGs with ERMES in yeast cells, we endogenously tagged Gem1, a potential regulatory ERMES subunit and a yeast ortholog of the metazoan mitochondrial Rho GTPase 1, Miro [67]. Gem1 protein is not an abundant protein in yeast cells since foci of the Gem1-TagRFP-T fusion protein were not easy to be distinguished from the background fluorescence. Nevertheless, microscopically observable Gem1-TagRFP-T foci were found associated with SGs decorated by Rpg1-3-GFP upon HS at 42 °C for 30 min (Figure 6B). Similar to experiments with Mmm1 and Mdm34, less frequent association of HS SGs with Gem1 was observed after HS at 46 °C.

These results unequivocally confirmed the association of heat stress-induced SGs in yeast cells with ER, mitochondria, and their contact sites ERMES.

### 2.5. Disassembly of Stress Granules after Heat Shock at 42 °C Is Also Hsp104-Dependent

Stress granules induced by HS at 46 °C have been found decorated by molecular chaperones [27,68], also participating in SGs disassembly. The disassembly of 46 °C-HS SGs was dependent on the main yeast disaggregase Hsp104 [27].

**Figure 7:**
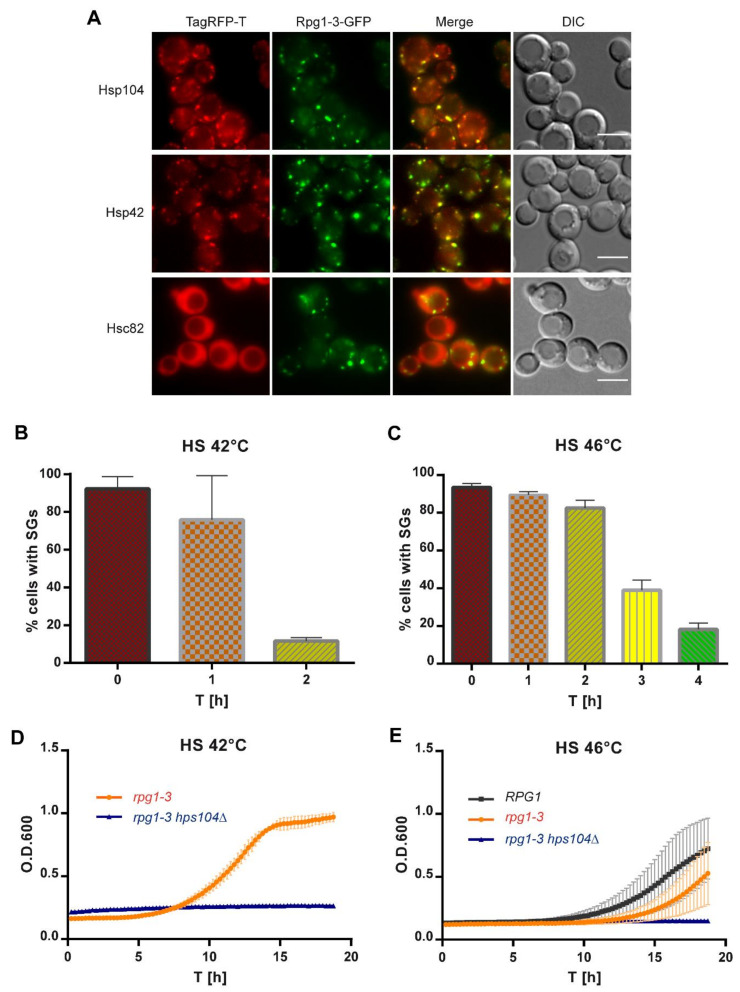
(**A**) Distribution of molecular chaperones upon HS at 42 °C. Live-cell imaging of exponentially growing cells incubated at 42 °C for 30 min producing Rpg1-3-GFP with TagRFP-T-tagged Hsp104, Hsp42, and Hsc82. Single focal planes of Z-stacks are presented. Scale bars, 5 µm. Disassembly of SGs after HS for 30 min at 42 °C (**B**) and 10 min at 46 °C (**C**) monitored as a percentage of cells in the population displaying Rpg1-3-labeled foci. (**D**) Regrowth of *rpg1-3*-*GFP* and *rpg1-3-GFP hsp104*Δ cells after 30 min-HS at 42 °C in YPD medium at 25 °C. (**E**) Regrowth of *RPG1-GFP*, *rpg1-3-GFP*, and *rpg1-3-GFP hsp104*Δ cells after HS for 10 min at 46 °C in YPD at 25 °C. Mean ± SD values for each measurement from two independent experiments were plotted in (**D**,**E**).

Therefore, we inspected SGs arising in *rpg1-3* cells upon HS at 42 °C for their association with molecular chaperones. As visible from Figure 7A, 42 °C-HS SGs were decorated by several heat shock proteins in double-labeled strains producing Rpg1-3-GFP and analyzed heat shock proteins as endogenous C-terminal TagRFP-T fusion proteins. Hsp104 colocalized with all visible SGs containing Rpg1-3-GFP and the same states for Hsp42. In contrast, yeast Hsp90 ortholog Hsc82 did not change its cellular distribution and stayed diffused in the cytoplasm and the nucleus upon HS at 42 °C. Association with molecular chaperones suggests that misfolded proteins are present within the stress granule the chaperones decorate. The presence of misfolded proteins might affect the dynamics of disassembly of such SGs that can be more difficult to resolve. Given the association of the main disaggregase with Rpg1-3-labeled SGs, we followed their disassembly after moderate and robust heat shock. After HS, a part of the cell suspension was immediately inspected by live-cell imaging, and the rest of the stressed culture was added to the fresh YPD medium and left to recover at 25 °C. Samples of recovering cells were taken in regular intervals and monitored by live-cell imaging for the disassembly of SGs. Cells in acquired images were manually counted using a Fiji plugin Cell Counter, and the percentage of cells with SGs in time was plotted (Figure 7B,C).

The disassembly of SGs in *rpg1-3* cells after the HS 42 °C at 25 °C took about two hours when only 12–14% of cells contained foci of Rpg1-3-GFP (Figure 7B). This number is similar to the percentage of cells displaying Rpg1-3-GFP foci at permissive growth conditions [57]. The disassembly of SGs in *rpg1-3* cells after the HS for 10 min at 46 °C was much slower, and only after 4 h of recovery at 25 °C, most of the cells displayed Rpg1-3-GFP diffused in the cytoplasm (Figure 7C). However, a part of the cells still contained Rpg1-3-GFP foci, which was similar to the recovery from HS at 42 °C. These Rpg1-3-GFP foci remaining in cells after both HS might represent SGs that were not cleared away during the recovery. Then, they would colocalize with the Hsp104 chaperone. Or, they are new foci raised due to the resumed translation. In this case, they should not be decorated with Hsp104 [57]. A recovery experiment with cells producing Rpg1-3-GFP and Hsp104-TagRFP-T showed that Hsp104-TagRFP-T did not accumulate on Rpg1-3-GFP foci remaining in cells after the recovery from HS at 42 °C (Figure S6A). This result suggests that cells dissolved SGs and resumed translation.

Next, we followed the regrowth of *rpg1-3* cells from HS at 42 °C and 46 °C and the effect of Hsp104 absence on it. As demonstrated in Figure 7D, *rpg1-3 hsp104*Δ cells did not recover the growth after HS at 42 °C during the time of monitoring. A similar result was obtained for this strain’s regrowth from HS at 46 °C (Figure 7E). Therefore, the recovery of *rpg1-3* cells from 42 °C-HS is dependent on Hsp104 chaperone similarly to 46 °C-HS. Although the resumption of growth of *rpg1-3 hsp104*Δ cells after HS at 42 °C and 46 °C is not visible from the presented data, both HS were not lethal for the strains with absent Hsp104, as documented by spot assays shown in Figure S6B,C. *rpg1-3* cells producing intact Hsp104 displayed a 3-h delay in the regrowth from 46 °C-HS compared to *RPG1 HSP104* cells. *rpg1-3* cells require more time to recover functional conformation of the Rpg1-3 protein and its interactions to resume translation and growth.

Our results show that the disassembly of SGs after moderate HS is also Hsp104-dependent, similar to that after robust HS. The high temperature destabilizes the Rpg1-3 mutant protein more than WT Rpg1, delaying the recovery from the stress.

### 2.6. Human TDP-43 Colocalizes with Heat Shock-Induced SGs in Yeast Cells and Facilitates Their Disassembly

Alterations in the dynamics of formation and disassembly of various mRNPs, including SGs, impact cell fitness and lifespan. They might also contribute to the pathophysiology of multiple diseases, including human neurodegenerative disorders [69,70,71,72]. We have selected proteins representing some of these disorders—TDP-43 (ALS, [73,74]), Htt103Q (Huntington’s disease [75]), and α-synuclein (Parkinson’s disease, [76])—to analyze their behavior in yeast cells under heat stress. All three human proteins were produced in yeast cells from the *GAL1* promoter upon induction with galactose. Their cellular distribution was followed by live-cell imaging after robust HS at 46 °C (Figure S7).

The Htt103Q-GFP fusion protein was found aggregated in one large spot per cell at unstressed conditions already, and its localization did not change after HS for 10 min at 46 °C (Figure S7A). Similarly, α-synuclein nicely decorating the cell periphery did not change its distribution upon the same HS conditions (Figure S7B). TDP-43 responded to robust HS as the only one from the three assayed proteins and changed its location from the nucleus to the cytoplasm. Although already aggregated in several large foci, TDP-43-GFP formed smaller foci after HS at 46 °C that overlapped with SGs formed by Rpg1-TagRFP-T (Figure S7C). The association of TDP-43 with SGs under HS has been reported for mammalian cells [44]. However, this is, to our knowledge, the first report on TDP43 colocalization with HS SGs in yeast cells.

**Figure 8:**
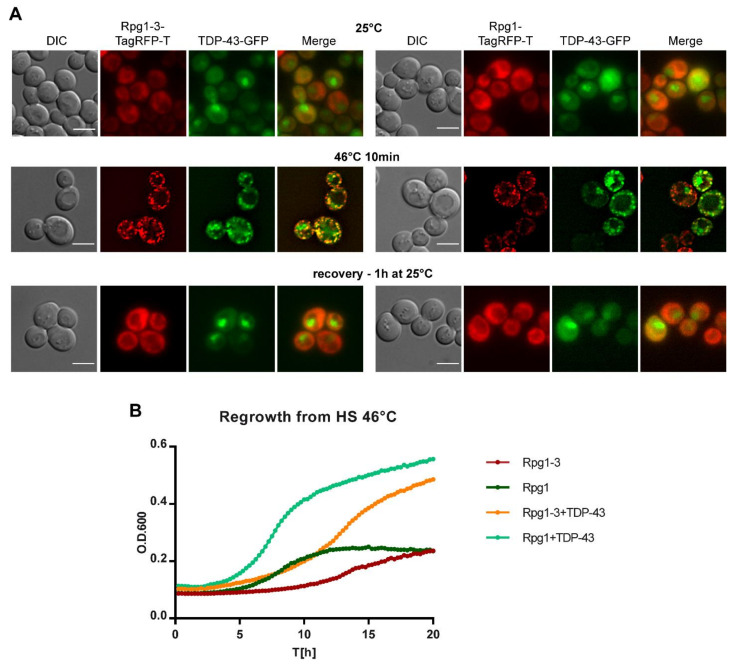
Cellular distribution of TDP-43-GFP in cells producing Rpg1-3-TagRFP-T and Rpg1-TagRFP-T. (**A**) Unstressed exponentially growing cells at 25 °C, after HS for 10 min at 46 °C and after the recovery at 25 °C for one hour. Single layers of Z-stacks are shown. Images after HS were deconvolved with the AMLE filter (Olympus). Scale bars, 5 µm. (**B**) Regrowth from HS at 46 °C in the glucose-based selective medium at 25 °C. A representative plot from three experiments with the same outcome is shown.

TDP-43 formed large aggregates even upon a short induction in the above experiment. It was probably due to its derepression during the overnight growth in the raffinose-containing medium before the induction with galactose. Therefore, we set up induction conditions in such a way to be able to follow an unaggregated protein under HS (see Section 4 for detailed experimental conditions). Briefly, cells exponentially growing in a selective glucose-based medium were transferred into a selective medium containing galactose. After 5-h induction, the culture was subjected to HS and then split. One part was analyzed by immediate live-cell imaging, and the rest of the culture was left to recover at 25 °C under shaking. Samples were taken regularly during recovery and inspected for the presence of TDP-43 foci in relation to SGs formed by Rpg1 or Rpg1-3. In unstressed cells, the TDP-43-GFP protein was accumulated in the nucleus and diffused in the cytoplasm (Figure 8A). No aggregates of TDP-43 were detected, confirming a correct setup of cultivation and induction conditions.

TDP-43 accumulated along with SGs upon HS for 10 min at 46 °C in both *rpg1-3* and *RPG1* cells (Figure 8A). Strikingly, after one hour of recovery at 25 °C in glucose-based medium, we observed no foci of either Rpg1-TagRFP-T or Rpg1-3-TagRFP-T. TDP-43-GFP neither formed any aggregates in the cytoplasm and could be seen accumulated back in the nucleus as in unstressed conditions. Although all SGs were disassembled already after one hour of recovery (Figure 8A), we continued the cultivation for one more hour with the same outcome, and no protein aggregates reappeared (not shown). In contrast, parental strains producing Rpg1-3-TagRFP-T and Rpg1-TagRFP-T only needed more time to disassemble SGs after HS at 46 °C. Rpg1-TagRFP-T-labeled SGs were disassembled after two hours of recovery at 25 °C, while Rpg1-3-TagRFP-T-labeled SGs were still detected in cells even after three hours of recovery at 25 °C (Figure S8). The distribution of TDP-43-GFP in cells harboring Rpg1-3-TagRFP-T or Rpg1-TagRFP-T upon HS at 42 °C for 30 min showed that TDP-43 was able to form distinguishable foci at 42 °C in cells with Rpg1-TagRFP-T that stayed diffused in the cytoplasm (Figure S7D). This observation indicates that human TDP-43, when produced in yeast cells, responds to heat stress independently of the formation of SGs. Nevertheless, TDP-43 colocalized with SGs formed by Rpg1-3-TagRFP-T at 42 °C (Figure S7D).

The surprisingly fast disassembly of SGs induced by robust HS upon the presence of TDP-43 was confirmed by monitoring the regrowth of cells from HS at 46 °C for 20 h. Parental strains producing Rpg1-TagRFP-T or Rpg1-3-TagRFP-T only needed much more time to restore their growth after HS at 46 °C compared to Rpg1-TagRFP-T and Rpg1-3-TagRFP-T strains producing TDP-43-GFP. The calculated time-shift indicated that parental *RPG1* cells reached OD600 = 0.3 four hours later than *RPG1* cells carrying TDP-43. *rpg1-3* cells needed even more time, seven hours, to get to the same OD600 value compare to the *rpg1-3 TDP-43-GFP* bearing cells. We repeated this experiment three times with the same outcome, always TDP-43 producing strains overgrew the parental strains.

Our data demonstrate that human TDP-43 associates with SGs formed upon heat shock in yeast cells and, unexpectedly, facilitates their disassembly and resumption of growth.

## 3. Discussion

In response to extracellular and intracellular stresses, cells form various mRNA–protein assemblies, including stress granules, to keep cellular protein homeostasis and modulate gene expression. Upon robust heat shock at 46 °C, yeast cells induce SGs decorated by the essential translation initiation factor eIF3a/Rpg1 [6,27]. We demonstrate here that the destabilization of Rpg1 within the PCI domain elicits the formation of SGs already at a moderate temperature of 42 °C when the intact Rpg1 stays evenly distributed in the cytoplasm. SGs formed at 42 °C in *rpg1-3* cells contain subunits of the eIF3 complex and other typical components of 46 °C-HS SGs such as mRNA, translation elongation, and termination factors, but not 40S and 60S ribosomal subunits. We have identified a novel component of yeast HS SGs, eIF2A/YGR054w, and we have also found out that both 42 °C- and 46 °C-HS SGs associate with ER and mitochondria and their contact sites ERMES. Similar to 46 °C-HS SGs, 42 °C-HS SGs are tightly associated with P-bodies, and their disassembly is Hsp104-dependent. Unexpectedly, the disassembly of HS SGs formed at 46 °C in yeast cells is facilitated by the presence of TDP-43, which is a human protein implicated in ALS pathologies.

The analyzed Rpg1 protein variant, Rpg1-3, carries a deletion and two amino acid substitutions in the PCI domain. Earlier, we have shown that it forms reversible assemblies without any applied stress linked to the exponential growth [57]. The Rpg1-3 assemblies containing other eIF3 subunits appear regularly in 15% of cells growing at 25 °C. Loss of Rpg1-3 function in translation during the cultivation at the non-permissive temperature of 37 °C does not lead to its additional aggregation. However, the Rpg1-3 protein accumulates in every cell when incubated for 30 min at 42 °C; when these conditions are intact, Rpg1 stays dominantly diffused in the cytoplasm. Rpg1-3 is not the only Rpg1 protein variant that responds to moderate HS at 42 °C. Rpg1-1, carrying a single amino-acid substitution in the C-terminal part of the protein [77], also accumulates in foci in every cell in the population after 30 min at 42 °C (Figure S2C). Experiments with Rpg1-1 performed parallelly to those with Rpg1-3 reported in this paper yielded similar results suggesting that the destabilization of eIF3a/Rpg1 leading to the translational arrest elicits the formation of SGs already at moderate HS, regardless of what part of Rpg1 is disturbed.

However, Rpg1 variants are not the only eIF3 subunits that accumulate at moderate HS. We have noticed an accumulation of C-terminally tagged eIF3b/Prt1 protein at 42 °C, leading to the accumulation of Rpg1 along with Prt1. We have not performed a more detailed analysis of the composition of Prt1 foci. However, seeing a coherent behavior of the subunits of the eIF3 complex that has been suggested earlier [18], we assume that Prt1 foci would behave similarly to SGs formed by the Rpg1-3 and Rpg1-1 variants. Although SGs have been so far thought to arise upon translational arrest due to the phosphorylation of eIF2α, interference with eIF4F, or inhibition of mTOR [3], the destabilization of eIF3 might be added to this list.

The absence of the 40S and 60S ribosomal subunits from SGs formed by Rpg1-3 and Rpg1 upon moderate and severe HS demonstrated in this work is in line with the results of several biochemical analyses published earlier [18,27,68,78]. The absence of 40S differs yeast SGs from SGs formed in metazoan cells, where stalled preinitiation complexes accumulate in SGs upon translational arrest and impaired translation initiation [3]. The reason might lie in different interactions within the yeast 48S preinitiation complex between translation initiation factors and the 40S subunit compared to mammalian one [79,80].

Stress granules are generally accepted to contain m^7^G-*capped* transcripts that become translationally silent, and other transcripts without the cap or having internal ribosome entry sites (IRES) are being translated by the translation machinery [81]. Interestingly, we have identified a new component of yeast HS SGs: a less-known protein eIF2A/YGR054w. This protein is an ortholog of the mammalian translation initiation factor eIF2A that functions in the AUG-dependent but GFP-independent recruitment of the 43S preinitiation complex and has been shown to suppress translation from IRES in *URE2* [60,82]. eIF2A/YGR054w is attractive, since its interacting proteins have been identified as suppressors and enhancers of the ALS-linked TDP-43 protein cytotoxicity in a large-scale screen performed in yeast [43].

The intriguing question is how SGs arise upon the translational arrest at 42 °C in *rpg1-3* cells? Is it an additional condensation of the translation machinery components on sites pre-determined by eEF3/Yef3 and Dcp2 [8]? Our results demonstrate that Yef3 is included in 42 °C-HS SGs that closely associate with Dcp2-labeled P-bodies similar to robust HS SGs [6]. However, we have noticed a different appearance of Yef3, eIF2A/YGR054w, eRF3/Sup35 foci being more concentrated in *rpg1-3* cells than in *RPG1* cells upon HS at 42 °C. These observations suggest a dominant regulation of concerned proteins distribution to SGs in *rpg1-3* cells induced by the translational arrest elicited by sequestration of Rpg1-3 from translation. Foci of Yef3, eIF2A/YGR054w, and Sup35 visible at 42 °C in *RPG1* cells might originate from Ded1-dependent phase separation and condensation occurring at this temperature [19]. Moreover, the Sup35 protein can undergo liquid–liquid phase separation (LLPS) [83]. RNA-helicase Ded1, reported as a “super-aggregator” [18], sequesters mRNAs with long and structured 5’ UTR upon 42 °C, thus causing a translational switch to allow for accurate stress response [19]. Indeed, LLPS [84] had already been accepted as a process underlying the assembly of macromolecular condensates, including stress granules, to ensure stress survival [85]. Thus, the observations of foci formed by destabilized proteins of the eIF3 complex mentioned above provoke us to speculate that they might arise due to thermally induced structural changes, leading to the phase separation and condensation of the concerned proteins. In this line, a script PSPer, an in silico screening tool for prion-like RNA-binding phase-separation proteins, assigned a high score to the human eIF3a [86]. All these data indicate that 42 °C-HS SGs in *rpg1-3* cells might be considered as condensates of proteins. However, we do not have enough experimental data to establish which signaling and regulatory pathways and platforms direct their formation.

Opinions promoting a pivotal role for RNA–RNA interactions in the condensation and recruitment of proteins to RNA condensate have emerged recently [21,78,87]. We observed an association of Rpg1- and Rpg1-3-labeled HS SG induced at 42 °C and 46 °C with ER and mitochondria. This finding suggests that a recently reported preference of the human eIF3 complex for mRNAs destined for translation at ER and mitochondria [88] might be valid also for the yeast eIF3 complex. Thus, the association of HS-induced SGs with ER and mitochondria and their contact sites ERMES indirectly points to a role of mRNAs in the formation of HS SGs in yeast cells.

Membranes of ER and mitochondria represent not only sites of membrane-associated translation but also deposition sites for misfolded proteins decorated by molecular chaperones [89,90]. Previously, the association of molecular chaperones with HS SGs at 46 °C has been shown [27,68,91]. We also noticed a need for active disaggregation during the recovery of yeast cells from moderate and robust HS, specifically for the functional disaggregase Hsp104, because its absence substantially delayed the regrowth of the cells after the stress relief. The colocalization of Hsp104 and Hsp42 chaperones with Rpg1-3-labeled HS SGs at 42 °C and 46 °C further suggested the presence of misfolded proteins within or associated with HS SG. The delayed disassembly of SGs formed by Rpg1-3 at 46 °C compared to Rpg1 further impinges on the Rpg1-3 protein itself as one of the putative clients of molecular chaperones. Hsp70 and Hsp40 chaperone families have been shown to affect the dynamics of Rpg1-3 assemblies arising at physiological growth conditions [57]. The involvement of other chaperones and components of the cellular protein quality network [92] next to Hsp104 emerges from the observation that neither moderate nor severe heat stress was lethal for yeast cells devoid of Hsp104. Our unpublished data indicate the presence of chaperones of the Hsp70 family Ssa1 and Ssa2 in HS SG formed by Rpg1-3 at 42 °C and 46 °C. These data comply with the previously suggested role for Hsp70/40 chaperones in the disassembly of stress granules [28].

Canonical stress granules are regarded as transiently arisen assemblies that are supposed to disassemble upon the stress relief [3] entirely, and biochemical analyses of heat-induced aggregation of the yeast [18] and human proteome [93] fully support this assumption. If aberrant SGs persist, cells have to cope with them by employing spatial protein quality control mechanisms that sequester such assemblies to deposition sites and/or degrade them by the ubiquitin–proteasome system (UPS) or by autophagy [34]. However, misfolded RNA-binding proteins and persistent mRNPs are also dangerous for cells, since they sequester proteins and mRNAs away from their sites of function.

Aberrant stress granules formation has been associated with neurodegenerative diseases [70,94], including ALS. ALS pathology-linked human nuclear protein TDP-43, which is implicated in RNA metabolism, was found to accumulate with stress granule markers in pathological brain tissues and cell lines after oxidative stress [95]. Since then, TDP-43 has been extensively studied relative to the formation of SGs as agents underlying pathological inclusions of the TDP-43 protein in the cytoplasm and nucleus. However, recent data point to a rather indirect relationship between the assembly of SGs and cytoplasmic aggregation of TDP-43 [47,48,49].

Our findings on facilitation of the disassembly of heat-induced SGs and recovery of yeast cells from HS by the presence of unaggregated TDP-43 are unique since, to our knowledge, there are no such studies suggesting a role of TDP-43 in SGs disassembly and stress recovery not only in yeast but also in mammalian cells. Given the speed of the disassembly of HS SGs and resumption of growth after the HS, we suggest that TDP-43 contributes to SGs disassembly by other mechanisms than molecular chaperones. The disassembly of heat-induced SGs is an energy-requiring process [6]. Refolding of unfolded and misfolded proteins within SGs is vital, as our above-discussed results on Hsp104 demonstrate. However, it is tempting to speculate that active processes involved in SGs disassembly concern not only proteins but also mRNAs sequestered in SGs and TDP-43, and its functions in RNA metabolism might be implicated either directly or indirectly.

This finding opens a new direction of research on the fate of SG-sequestered mRNAs in relation to persistent accumulations of SG markers found in pathologies of many neurodegenerative disorders. We propose eIF3a/Rpg1 and its variants forming HS SGs with altered dynamics as a novel model for such studies.

## 4. Materials and Methods

### 4.1. Strains and Growth Conditions

The *Saccharomyces cerevisiae* strains used in this study are listed in Table S1, plasmids are listed in Table S2, and primers are listed in Table S3. *Escherichia coli* DH5α [*F− rec A1 supE44 endA1 hsdR17 (rk−, mk+) gyrA96 relA1 thi-1* ∆*(lacIZYA-argF)U169deoR* (Φ*80d*∆ *(lacZ) M15*] was used as a host in cloning procedures. Standard bacterial cultivation media and temperatures were used, if not stated otherwise. Standard methods were used for all DNA manipulations [96]. Yeast cell transformation was carried out according to the standard protocols [97], and when needed, antibiotics hygromycin (Invivogen, Toulouse, France) at 300 μg/mL, nourseothricin (Werner Bioagents, Jena, Germany) at 100 µg/mL, and geneticin (Gibco/ThermoFisher Scientific, Waltham, MA, USA) at 400 µg/mL were used for selection of transformants.

*S. cerevisiae* strains were grown either in the standard rich medium (YPD) or synthetic defined media supplemented with drop-out mixtures missing appropriate amino acids and 2% glucose (SD media). Media for induction of the *GAL1* promoter contained 2% galactose (SC-galactose media). Yeast thermosensitive mutants were cultivated at 25 °C and wild-type strains were cultivated at 30 °C. Low-fluorescence synthetic medium, LF-SC-trp was used for live-cell imaging experiments. LF-SC-trp containing 2% glucose and trp- drop-out was based on low-fluorescence YNB (LF-YNB, Formedium, UK).

#### 4.1.1. Galactose-Induction Conditions

Analyzed strains were grown overnight in glucose-containing selective media to saturation and back-dilution into a fresh selective medium to reach OD600 = 0.5–0.6, corresponding to the middle exponential growth phase the following day. The next day, the appropriate volume of cells that would give OD600 = 0.5–0.6 in the needed volume of an inducing media was taken and washed to remove traces of glucose. The cell pellet was resuspended in an appropriate selective-galactose-containing medium, in which cells were further incubated for 5 h to induce expression of the analyzed protein. After 5-h induction with galactose, culture volumes were OD600 normalized, centrifuged, and resuspended in 1 mL of a medium prewarmed to 46 °C and further incubated at 46 °C for 10 min with agitation in an Eppendorf Thermomixer. After heat shock, the culture was split. One part was subjected to immediate live-cell imaging, and the rest of the culture was let to recover at 25 °C under shaking.

#### 4.1.2. Regrowth after Heat Shock

To follow the regrowth from the stress, heat-shocked cells were transferred to the appropriate medium in a 6- or 12-well plate and placed into BioTek-EON^TM^ microplate spectrophotometer. The absorbance at 600 nm was recorded every 15 min. Time-shift in reaching OD600 = 0.3 between analyzed cultures was calculated according to an earlier described protocol [98]. Graphs were prepared in GraphPad Prism6 (GraphPad Software, La Jolla, CA, USA).

### 4.2. Construction of Plasmids and Yeast Strains

Plasmids used and constructed in this study can be found in Table S2 and primers can be found in Table S3. The plasmid-derived Rpg1-3-yTagRFP-T (pIM23-Rpg1-3 [*HIS3*]) and Rpg1-yTagRFP-T (pIM23-Rpg1 [*HIS3*]) fusions were created in the pIM23 vector [64] by *Sac*I and *Xba*I subcloning of the *rpg1-3* and *RPG1* coding sequences under the native *RPG1* promoter from the plasmids pRpg1-3-GFP [*HIS3*] and pRpg1-GFP [*HIS3*] [57], respectively. Ligated products were transformed into *E. coli* and analyzed by colony PCR and sequencing.

To construct strains carrying either Rpg1-3-yTagRFP-T (CRY2410) or Rpg1-Tag-RFP-T (CRY2412) fusions on a plasmid as the only copy of the essential *RPG1* gene covering its deletion in the chromosome, a procedure described previously has been used [57].

Single-, double-, or triple-labeled strains with endogenous C-terminal yTagRFP-T or ymTagBFP fusions were created by the integration of PCR-amplified cassettes carrying yTagRFP-T (pIM690, pIM700) or ymTagBFP (pIM573), as described previously [64]. The correct integration of cassettes was confirmed by colony PCR. Some double-labeled strains were generated by mating and subsequent sporulation. Spore dissection was performed on a Singer^TM^ micromanipulator. All fluorescently labeled strains were checked by Western blotting with corresponding antibodies.

### 4.3. Western Blotting

NaOH/TCA protein extracts [99] were prepared and subjected to SDS-PAGE. Resolved proteins were transferred to PROTRAN nitrocellulose membrane (Sigma-Aldrich/Merck, Darmstadt, Germany) or PVDF (Sigma-Aldrich/Merck, Darmstadt, Germany). Membranes were blocked with 5% non-fat milk (Regilait, Saint-Martin-Belle-Roche, France) and probed with corresponding antibodies. Red fluorescent protein fusions were detected with an anti-tRFP antibody (AB233, Evrogen, Moscow, Russia) that recognizes TagRFP-T and mTagBFP, and with an anti-RFP antibody (3F5, Chromotek, Planegg-Martinsried, Germany) recognizing mRFP and mCherry fluorescent proteins. The antibodies were used at dilution 1:1000 at 4 °C overnight. As a secondary antibody, the goat anti-rabbit IgG antibody conjugated to HRP (32460, Pierce-ThermoFisher Scientific, Waltham, MA, USA) and the goat anti-mouse IgG antibody conjugated to HRP (32430, Pierce-ThermoFisher Scientific, Waltham, MA, USA) were used both at dilution 1:600. GFP protein fusions were assayed with the GFP(B-2) HRP conjugate (sc-9996, Santa Cruz Biotechnology, Dallas, TX, USA) at dilution 1:1000, 1 h at RT or overnight at 4 °C. Proteins were detected by SuperSignal West Dura Substrate (Pierce-ThermoFisher Scientific, Waltham, MA, USA).

### 4.4. Differential Centrifugation

Strains were cultivated in YPD at 25 °C to exponential phase, heat-shocked at 42 °C for 30 min, and then washed and resuspended in the lysis buffer containing 50 mM Tris-HCl (pH 7.6), 50 mM NaCl, 5 mM MgCl_2_, 0.1% NP-40, 1 mM β-mercaptoethanol, 1 mM PMSF, 10 mM NEM, and Complete Mini EDTA-free Protease Inhibitor Mix (Roche, Basel, Switzerland). The disruption of cells and centrifugations were carried out as described in [54]. The resulting pellets and supernatants were analyzed by SDS-PAGE and Western blotting with specific antibodies. Antibodies against eIF3 subunits were a kind gift from L. Valasek (IMIC, Prague, Czech Republic) and were used at the following dilutions—rabbit anti-Hcr1 (1:1000), rabbit anti-Nip1 (1:500), rabbit anti-Prt1 (1:1000), and rabbit anti-Tif35 (1:3000). Small and large ribosomal subunits were probed with rabbit anti-Rps0A (1:500, L. Valasek, IMIC, Prague, Czech Republic) and rabbit anti-Rpl15 (1:1000, ab155802, Abcam, Cambridge, UK). Rpg1-GFP and Rpg1-3-GFP were probed with an anti-GFP antibody (1:1000, sc-9996, Santa Cruz Biotechnology, Dallas, TX, USA) and Pgk1 with anti-Pgk1 antibody (1:10,000, ab113687, Abcam, Cambridge, UK).

### 4.5. Polysome Profile Analyses

Cells were grown to an OD600 ~ 1, heat-shocked at 42 °C for 30 min or 46 °C for 10 min, and cycloheximide (CHX, Sigma-Aldrich/Merck, Darmstadt, Germany) was added to the culture to a final concentration of 50 μg/mL 5 min before harvesting. The detailed protocol has been described previously [57].

### 4.6. Live-Cell Microscopy and Image Analysis

Prior to imaging, cells were washed off the growth or heat-shock medium with a low-fluorescence synthetic medium, mounted on a cover glass, and coated with a thin slice of 1.5% agarose prepared in LF-SC-trp. Widefield microscopy was performed using an Olympus IX-81 inverted microscope equipped with a 100× PlanApochromat oil-immersion objective (NA = 1.4) and a Hamamatsu Orca-ER-1394 digital camera. Fluorescence was detected with appropriate filter blocks—ymTagBFP with the BFP filter block exc. 390/18 nm, em. 435–485 nm; GFP with the filter block U-MGFPHQ, exc. 460–488 nm, em. 495–540 nm; DsRed, RFP and yTagRFP-T with the CY3HQ filter block exc. 545/30 nm, em. 610/75 nm. Z-stacks were taken with 0.2 µm z-steps, and if needed, deconvolution with the Advanced Maximum Likelihood (AMLE) filter (Xcellence Imaging SW, Olympus, Tokyo, Japan) was performed. Images were collected in 16-bit format, processed using Olympus Cell-R^TM^ Xcellence software (Olympus, Tokyo, Japan), and Fiji/ImageJ2 (NIH, Bethesda, MD, USA) [100]. Cropped images were adjusted for brightness/contrast and mounted in Adobe Illustrator (Adobe, San Jose, CA, USA). Single focal planes of Z-stacks are presented in all figures, and deconvolution usage is stated in the figure caption. Representative microscopic images from more than two experiments with the same outcome are shown.

### 4.7. Image Analysis and Quantification

To monitor the colocalization of analyzed proteins in microscopic images, plot profiles of fluorescence intensity along a selected line were prepared using a Fiji macro “Line Intensity Profile” (Imperial College, London, UK). The macro creates a line intensity profile plot and data table of a multichannel image. Graphical presentations were prepared using GraphPad Prism6 (GraphPad Software, La Jolla, CA, USA).

To quantify the various aspects of accumulations arising in yeast cells, we participated in the development of a custom-written script named oCellaris that is capable of semi-automated granulometry analysis of microscopic images. It identifies cells in the bright-field image and computes cell contours. A set of algorithms enables fitting fluorescence images to cell contours and computing the various fluorescence parameters of each cell in the analyzed image. The calculated parameters allow us to evaluate quantitatively and qualitatively fluorescence in acquired images and compare cells within a population or between strains. The script also enables a graphical presentation of analyzed images such as the 3D-brightness displayed in Figure 1C and Figure S2D.

## 5. Conclusions

Translation as one of the key steps in gene expression is also an important player in maintaining proteostasis in cells of all living organisms, including humans. Any impairment of this fundamental process might have serious consequences. Mutations in genes encoding components of the translation machinery and regulatory factors have been linked to several neurodevelopmental disorders. The human translation initiation factor subunit eIF3a, which is a constituent of many types of SGs, has been found aggregated in neutrophils isolated from the blood of Alzheimer’s patients. Yeast eIF3a/Rpg1 destabilized within the PCI domain is vulnerable to aggregation at physiological growth conditions. The destabilization of Rpg1 leads to altered dynamics of heat shock-induced stress granules, earlier formation, and delayed disassembly. HS SGs labeled with Rpg1, containing mRNAs and components of the translation machinery, associate with endoplasmatic reticulum, mitochondria, and their contact sites ERMES. Significantly, the disassembly of yeast HS SGs is facilitated by the presence of TDP-43, which is a human protein implicated in ALS. We believe that our results obtained on the yeast model will be instrumental to studies in metazoan cells to deepen our understanding of the role of the translation machinery and its components in neurodegeneration.

## Data Availability

Not applicable.

## Supplementary Materials

The following are available online at https://www.mdpi.com/article/10.3390/ijms22105164/s1, Figure S1: Biochemical and microscopic analysis of WT Rpg1-GFP and mutant Rpg1-3-GFP under robust heat shock, Figure S2: Cellular distribution of eIF3 subunits in *RPG1* and *rpg1**-1* cells upon moderate heat shock, Figure S3: Cellular distribution of Yef3, Sup35, or eIF2A/YGR054w upon heat shock, Figure S4: Cellular distribution of mRNAs and Dcp2 in cells with Rpg1-3-GFP, Figure S5: Distribution of 46 °C-HS SGs of Rpg1 relative to ER and mitochondria, Figure S6: Heat shock recovery and survival, Figure S7: Cellular distribution of Htt103Q-GFP, α-synuclein-RFP, and TDP-43-GFP upon heat shock, Figure S8: Recovery of strains producing Rpg1-3-TagRFP-T and Rpg1-TagRFP-T after HS at 46 °C, Table S1: Yeast strains used in this study [101], Table S2: Plasmids used in this study, Table S3: Oligonucleotides used in this study, Video S1: Movement of Rpg1-3-GFP-labeled SGs in association with Dcp2-TagRFP-T after HS at 42 °C [102].
