# Supplementary material for: eIF3a Destabilization and TDP-43 Alter Dynamics of Heat-Induced Stress Granules
Source: Int J Mol Sci. 2021 May 13;22(10):5164. doi: 10.3390/ijms22105164 (PMC8153170; doi:10.3390/ijms22105164)

Figure S4. Cellular distribution of mRNAs and Dcp2 in cells with Rpg1-3-GFP. (**A)** Live-cell imaging of exponentially growing cells heat-shocked at 46°C for 10 min producing Rpg1-3 and fluorescently-labeled mRNAs - *ENO2*-mCherry, *ASH1*-GFP, and *DOA1*-GFP. After deconvolution with the AMLE filter (Xcellence software, Olympus), single representative layers of Z-stacks are presented (**B**). Live-cell imaging of cells carrying Rpg1-3-GFP and Dcp2 endogenously tagged with TagRFP-T growing exponentially at 25°C. Single layer of a Z-stack is shown. Scale bars, 5μm.

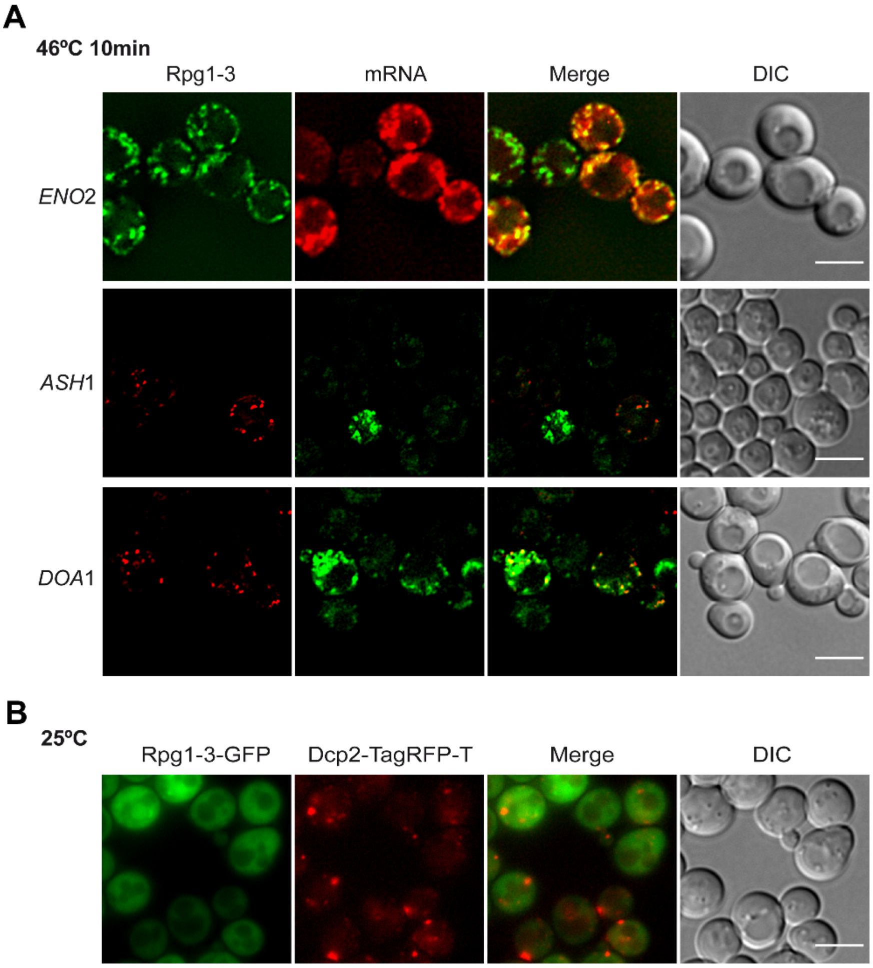

Supplement: Supplementary file 1 [file ijms-22-05164-s001.zip › Malcova et al Figure S4.pdf]
