# Supplementary material for: eIF3a Destabilization and TDP-43 Alter Dynamics of Heat-Induced Stress Granules
Source: Int J Mol Sci. 2021 May 13;22(10):5164. doi: 10.3390/ijms22105164 (PMC8153170; doi:10.3390/ijms22105164)

Figure S5. Distribution of 46°C-HS SGs of Rpg1 relative to ER and mitochondria. Live-cell imaging of exponentially growing cells heat-shocked at 46°C for 10 min carrying Rpg1-GFP with (**A**) mito-RFP (mitochondrial marker) or (**B**) HDEL-DsRed (ER marker). Single representative layers of Z-stacks after deconvolution with the AMLE filter (Xcellence software, Olympus) are presented. Scale bars, 5µm.

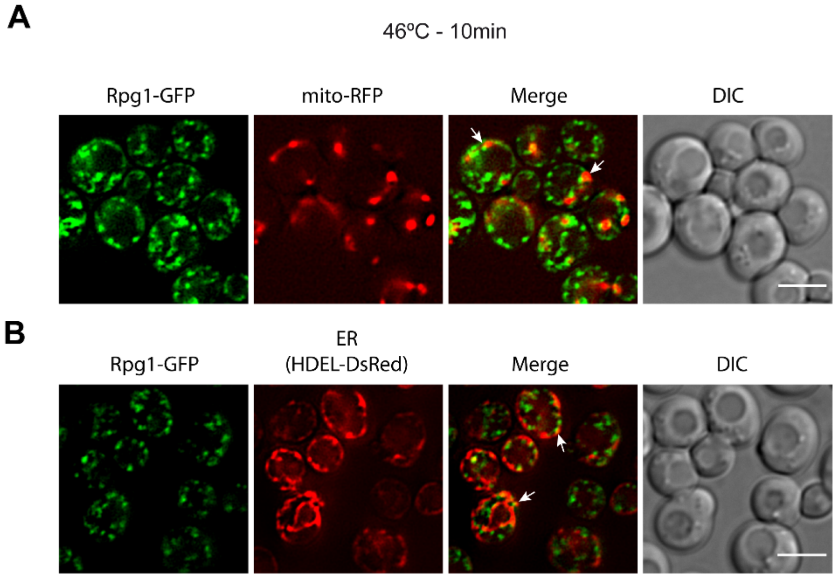

Supplement: Supplementary file 1 [file ijms-22-05164-s001.zip › Malcova et al Figure S5.pdf]
