# Supplementary material for: eIF3a Destabilization and TDP-43 Alter Dynamics of Heat-Induced Stress Granules
Source: Int J Mol Sci. 2021 May 13;22(10):5164. doi: 10.3390/ijms22105164 (PMC8153170; doi:10.3390/ijms22105164)

Figure S6. Heat shock recovery and survival. (**A**) Cells carrying Rpg1-3-GFP and Hsp104 endogenously tagged with TagRFP-T after 2 h of recovery from heat shock for 30 min at 42°C. Single representative layers of Z-stacks are presented. Scale bar, 5μm. Spot assays of Rpg1-3-GFP producing cells with Hsp104 and *hsp104Δ* after HS at 42°C for 30 min (**B**) or (**C**) Wild-type cells or cells with the deletion of *hsp104* after HS at 46°C for 10 min incubated for two days at 25°C (**B**) and 30°C (**C**).

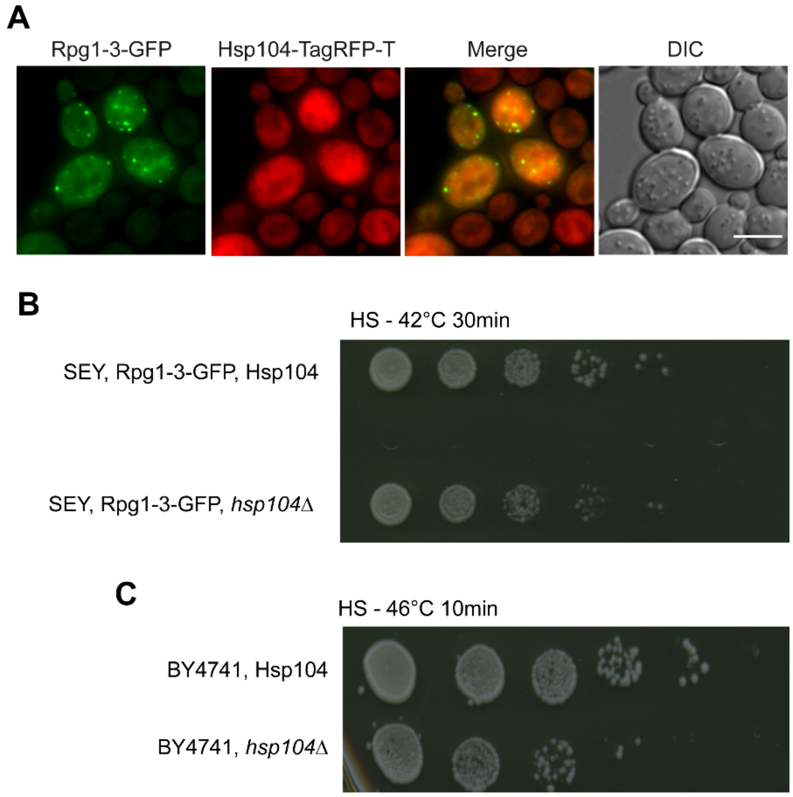

Supplement: Supplementary file 1 [file ijms-22-05164-s001.zip › Malcova et al Figure S6.pdf]
