# Supplementary material for: eIF3a Destabilization and TDP-43 Alter Dynamics of Heat-Induced Stress Granules
Source: Int J Mol Sci. 2021 May 13;22(10):5164. doi: 10.3390/ijms22105164 (PMC8153170; doi:10.3390/ijms22105164)

Figure S7. Cellular distribution of Htt103Q-GFP, α-synuclein-RFP, and TDP-43-GFP upon heat shock. Live-cell imaging of (**A**) Htt103Q-GFP, (**B**) α-synuclein-RFP, and (**C**) TDP-43-GFP in strains carrying Rpg1. Unstressed cells cultivated at 30°C in the galactose-inducing selective medium and after the heat shock for 10 min at 46°C. (**D**) Distribution of TDP-43-GFP in cells harboring Rpg1-3-TagRFP-T or Rpg1-TagRFP-T heat-shocked at 42°C for 30 min. Single representative layers of Z-stacks are presented. Scale bars, 5μm.

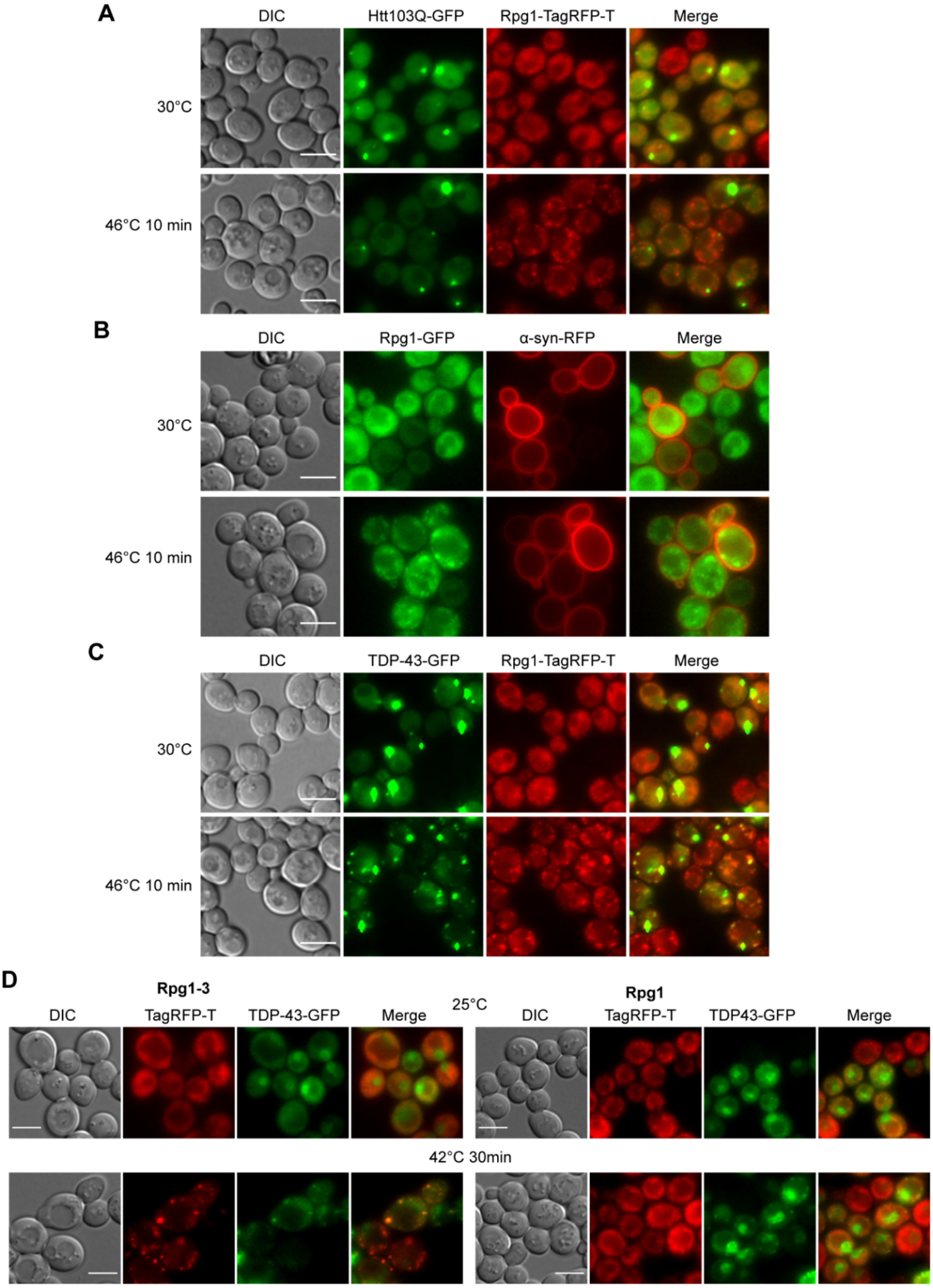

Supplement: Supplementary file 1 [file ijms-22-05164-s001.zip › Malcova et al Figure S7.pdf]
