# Supplementary material for: eIF3a Destabilization and TDP-43 Alter Dynamics of Heat-Induced Stress Granules
Source: Int J Mol Sci. 2021 May 13;22(10):5164. doi: 10.3390/ijms22105164 (PMC8153170; doi:10.3390/ijms22105164)

Figure S8. Recovery of strains producing Rpg1-3-TagRFP-T and Rpg1-TagRFP-T after HS at 46°C. Live-cell imaging of unstressed cells cultivated at 30°C in the galactose-inducing selective medium, after the heat shock for 10 min at 46°C and during the recovery from the HS in the YPD medium at 25°C. Single representative layers of Z-stacks are presented. Scale bars, 5μm.

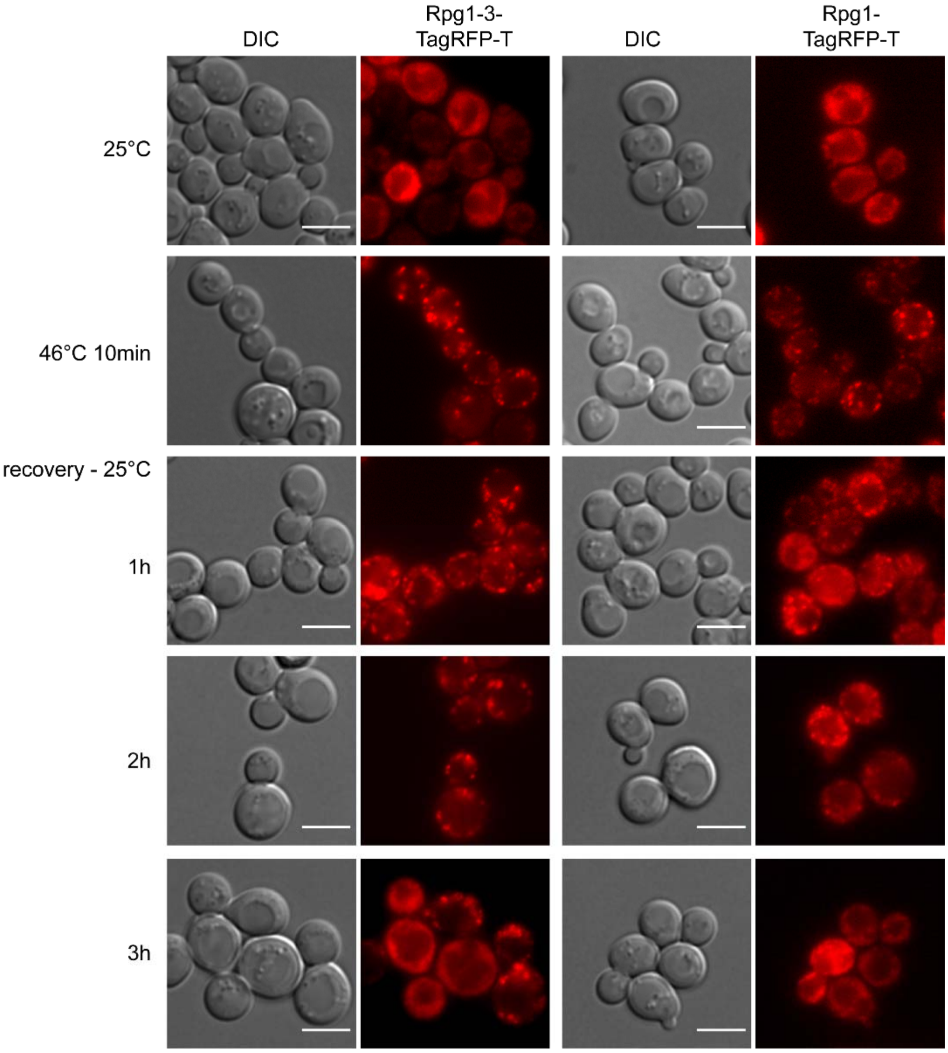

Supplement: Supplementary file 1 [file ijms-22-05164-s001.zip › Malcova et al Figure S8.pdf]
