# Supplementary material for: eIF3a Destabilization and TDP-43 Alter Dynamics of Heat-Induced Stress Granules
Source: Int J Mol Sci. 2021 May 13;22(10):5164. doi: 10.3390/ijms22105164 (PMC8153170; doi:10.3390/ijms22105164)
Supplement: Supplementary file 1 [file ijms-22-05164-s001.zip › Malcova et al. - Supplementary Tables S1-S3-03052021.pdf]

Table S1. Yeast strains used in this study

Strain	Genotype	Reference
CRY155	BY741; *MATa; his3Δ1; leu2Δ0; met15Δ0; ura3Δ0*	Euroscarf
CRY257	SEY6210 *MATα NIP1-mRFP::kanMX4*	[57]
CRY307	BY4741 *hsp104::kanMX4*	Euroscarf
CRY1280	YMK1587; *MATα ADE2 his3-11,15 leu2-3,112 trp1-1 ura3-1 ENO2-MS2L,*pMS2-mCh$_3$ [*HIS3*]	[13]
CRY1661	SEY6210, *MATα leu2-3,112 ura3-52 his3-Δ200 trp1-Δ901 suc2-Δ9 lys2-801* [PIN^{+}] [PSI^{-}]	[101]
CRY1662	SEY6210.1; *MATa leu2-3,112 ura3-52 his3-Δ200 trp1-Δ901 suc2-Δ9 lys2-801* [PIN+]	[101]
CRY1679	SEY 6210.1 *rpg1Δ::natNT2* + pRPG1-3-GFP [*HIS3*] [PSI^{+}] [PIN^{+}]	[57]
CRY1681	SEY 6210.1 *rpg1Δ::natNT2* + pRPG1-1-GFP [*HIS3*] [PSI^{+}] [PIN^{+}]	[57]
CRY1683	SEY 6210.1 *rpg1Δ::natNT2*+ pRPG1-GFP [*HIS3*] [PSI^{+}] [PIN^{+}]	[57]
CRY1861	CRY1679 *HSP104-yTagRFP-T::hphNT1*	[57]
CRY1868	CRY1679 *DCP2-y-TagRFP-T::hphNT1*	[57]
CRY1873	CRY1679 *HCR1-yTagRFP-T::hphNT1*	[57]
CRY1874	CRY1679 *PRT1-yTagRFP-T::hphNT1*	[57]
CRY1888	*CRY1679xCRY257; rpg1Δ::natNT2* + pRPG1-3-GFP [*HIS3*] *NIP1-mRFP::kanMX4*	[57]
CRY1889	*CRY1683xCRY257; rpg1Δ::natNT2* + pRPG1-GFP [*HIS3*] *NIP1-mRFP::kanMX4*	
Y1897	CRY1679 *HSP42-yTagRFP-T::hphNT1*	[57]
CRY1901	CRY1679 *HSC82-yTagRFP-T::hphNT1*	[57]
CRY1905	CRY1679 *HSP82-yTagRFP-T::hphNT1*	[57]
CRY1934	CRY1683 *trp1-Δ901::HDEL-DsRed::TRP1*	This study
CRY1936	CRY1679 *trp1-Δ901::HDEL-DsRed::TRP1* + pYX142-mito-ymTagBFP [*LEU2*]	[57]
CRY1942	CRY1683 + pYX142-mito-mRFP [*LEU2*]	This study
CRY1949	CRY1681 *HCR1-yTagRFP-T::hphNT1*	This study
CRY1950	CRY1681 *PRT1-yTagRFP-T::hphNT1*	This study
CRY2018	*CRY1679xCRY2072, rpg1Δ::natNT2* + pRPG1-3-GFP [*HIS3*] *YEF3-yTaqRFP-T::hphNT1*	[57]
CRY2020	CRY1683 *YEF3-yTaqRFP-T::hphNT1*	This study
CRY2024	CRY1683 *HCR1-yTagRFP-T::hphNT1*	This study
CRY2025	CRY1683 *PRT1-yTagRFP-T::hphNT1*	This study
CRY2028	SEY6210 *PRT1-yTagRFP-T::hphNT1*	This study
CRY2035	CRY1679 *RPS30A-yTagRFP-T::hphNT1*	This study

CRY2048	CRY1679xCRY2051, Sup35-yTagRFP-T/ Sup35 SEY2n *Δrpg1/RPG1* + Rpg1-3-GFP	This study
CRY2050	CRY1683xCRY205,Sup35-yTagRFP-T/ Sup35 SEY2n *Δrpg1/RPG1* + RPG1-GFP	This study
CRY2051	SEY 6210 *SUP35*-yTagRFP-T*::hphNT1*	This study
CRY2072	SEY6210 *YEF3-yTagRFP-T::hphNT1*	This study
CRY2137	CRY1681xCRY257*; rpg1Δ::natNT2*+ pRPG1-1-GFP [*HIS3*] *NIP1-mRFP::kanMX4*	This study
CRY2167	CRY1683 *YGR054w-yTagRFP-T::hphNT1*	This study
CRY2169	CRY1679 *YGR054w-yTagRFP-T::hphNT1*	This study
CRY2295	CRY1679 *XRN1-yTagRFP-T::hphNT1*	[57]
CRY2315	CRY1280xCRY2084; *rpg1Δ::natNT2* + pUG35-RPG1-3 *[URA3] ENO2-MS2L,*pMS2-mCh3 *[HIS3]*	[57]
CRY2336	CRY1679 *trp1-Δ901::HDEL-DsRed::TRP1*	[57]
CRY2337	CRY1679 + pYX142-mitoRFP [*LEU2*]	[57]
CRY 2353	CRY1683 *trp1-Δ901::HDEL-DsRed::TRP1*	
CRY2410	SEY; MAT*α ura3-52 his3-Δ200 rpg1Δ::natNT2*+ pIM23-RPG1-3-TagRFP-T [*HIS3]*	This study
CRY2412	SEY; *MAT*a *ura3-52 his3-Δ200 rpg1Δ::natNT2*+ pIM23-RPG1-TagRFP-T [*HIS3]*	This study
CRY2508	CRY1679 *hsp104Δ::kanMX4*	[57]
CRY2660	CRY1679 *GEM1-yTagRFP-T::hph1NT1*	This study
CRY2731	YET288, BY4741, *MATa;his3Δ1; leu2Δ0; met15Δ0; ura3Δ0 ASH1::ASH1*-24XMS2V6WT *kan-*	[62]
CRY2733	YET345, BY4741, *MATa;his3Δ1; leu2Δ0; met15Δ0; ura3Δ0 DOA1::DOA1*-24XMS2V6WT *kan-*	[62]
CRY2761	CRY2410 + pGAL1-GFP-TDP43 [*URA3*]	This study
CRY2763	CRY1683 + pGAL1-GFP-TDP43 [*URA3*]	This study
CRY2767	YET288, BY4741 *MATa;his3Δ1; leu2Δ0; met15Δ0; ura3Δ0 ASH1::ASH1*-24XMS2V6WT *kan-*, pET296 [*LEU2*], pIM35-Rpg1-3 [*URA3*]	[57]
CRY2799	CRY1679 *MMM1-yTaqgRFP-T::hph1NT1*	This study
CRY2831	CRY1679 *MMM1-yTagRFP-T::hph1NT1, MDM34-ymTagBFP-kanMX6*	This study
CRY2842	YET345 BY4741, *MATa;his3Δ1; leu2Δ0; met15Δ0; ura3Δ0 DOA1::DOA1*-24XMS2V6WT *kan-* , pET296 [*LEU2*], pIM35-Rpg1-3 [*URA3*]	[57]
CRY2988	CRY1683 + pα-Syn-RFP [*URA3*]	This study

Table S2. Plasmids used in this study

Plasmid	Description	Reference/Source
pIM690	pFA6a-yTagRFP-T-hphNT1, AmpR	[64]
pIM700	pFA6a-yTagRFP-T-NatNT2, AmpR	[64]
pIM573	pFA6a-ymTagBFP-kanMX4, AmpR	[64]
pUG23	CEN/ARS, MET25p, EGFP3, HIS3, AmpR	J. Hegeman lab
pRPG1-3-GFP	pUG23, CEN/ARS, Rpg1p-Rpg1-3-yEGFP3, HIS3, AmpR	[57]
pRPG1-1-GFP	pUG23,CEN/ARS, Rpg1p-Rpg1-1-yEGFP3, HIS3, AmpR	[57]
pRPG1-GFP	pUG23,CEN/ARS, Rpg1p-Rpg1-yEGFP3, HIS3, AmpR	[57]
pIM23	CEN/ARS, MET25p, yTagRFP-T, HIS3, AmpR	[64]
pIM23-Rpg1-3	CEN/ARS, Rpg1p-Rpg1-3-yTagRFP-T, HIS3, AmpR	This study
pIM23-Rpg1	CEN/ARS, Rpg1p-Rpg1-yTagRFP-T, HIS3, AmpR	This study
HDEL-DsRed	YIplac204TKC-DsRed-Express2-HDEL, TRP1, AmpR	B. Glick lab
pYX142-mitoRFP	CEN/ARS, TPI1p, LEU2, replacement of GFP with mRFP	J. Shaw lab
pUG35-RPG1-3-GFP	pUG35, CEN/ARS, pRpg1-Rpg1-3-EGFP3, URA3, AmpR	[57]
Htt103Q-GFP	pCu426, GAL1p-Htt103Q-GFP, URA3, AmpR	[102]
TDP-43-GFP	pCu426, GAL1p-TDP-43-GFP, URA3, AmpR	[102]
α-Syn-RFP	pRS426 GAL1p, α-Synuclein-RFP, URA3, AmpR	J. Hasek lab

Table S3. Oligonucleotides used in this study

Name	Sequence 5´- 3´
RPGgfp3	AAATCTAGACCTGCCCCCCTTGG
RPGgfpc5	AGTGAATTCGAGCTCAACTTGATG
Hsp104trfp5	GATGACGATAATGAGGACAGTATGGAAATTGATGATGACCTAGATCTGA AGCTTCGTACGCTGCAG
Hsp104C2	CTGATTCTTGTTCGAAAGTTTTTAAAAATCACACTATATTAAACACTATA GGGAGACCGGCAG
HSP104 control	ACCAAGAATTTGGTCATGGG
Dcp2mc5	CGAATGGAACTTCAGGGTCTAATGAATTATTAAGCATTTTGCATAGGAA GCGTACGCTGCAGGTCGAC
Dcp2mc3	CGGCTGCCTTCATTTACAGTGTGTCTATAAAACGTATAACACTTATTCTTA TCGATGAATTCGAGCTCG
Dcp2 control	CAAGCGAACTTTTGGGAATG
Hcr1C5	CGACCTAGATGGCCCCGACGATTTTGAATTCGGTGATGACGACTTTATG CTTCGTACGCTGCAGGTCGA
Hcr1C3	CAACAAAATGAGATGGACAAGTTTATCATAGCAAAGAAACAATAAGCA GACACTATAGGGAGACCGGCAG
Hcr1Cdiafw	CCGTGTTAGAGGTGGTACTG
Prt1C5	CGAAGAGATAGTTGAAGAAGTTTTGGAAGAAACAAAGGAAAAGGTCGA ACTTCGTACGCTGCAGGTCGA
Prt1C3	CTTATACTAGATATTGTTGTTAGTTTACATACAAGCCCGTAAGTTCATGA AATCACTATAGGGAGACCGGCAG
PrtFor1696	TTAGTGGGTCCAAACATGCGTAGAT
NipRFPfor	CGCTAAAGAACGTCTTAACCCACCATCAAATCGTCGTGGAGCAGGGGCG GGTGC
NipRFPrev	TTGTTAAGAATGCTTTCGAAGTAGTTTTTGATTTTATTGATCCCCCTCGAG GTCGACGGTATCG
NipFor1952	AAAATCCATTCGTCGTTCCTTAG
Hsp26C5	GAACCACGTCAAGAAGATTGAGGTTTCTTCTCAAGAATCGTGGGGTAAC CTTCGTACGCTGCAGGTCGA
Hsp26C3	CAAGCAACAATGGTCCTCGCGAGAGGGACAACACTATAGAGCCAGGTC ACTCACTATAGGGAGACCGGCAG
Hsp26Cdiafw	GAAAGTCGTGGTTCCTGGTGTC
Hsp42C5	CCGACGAAGAATTGGAGTTTGAAGAAAATCCCAACCCTACGGTAGAAA ATCTTCGTACGCTGCAGGTCGA
Hsp42C3	GCTTATTATAAATATAAATGTATGTATGTGTGTATAAACAGATACGATAT CACTATAGGGAGACCGGCAG

Hsp42Cdiafw	CCGAGGACACTTACGTAGTTG
Hsc82C5	CCAGTTGAAGAGGTTCCAGCTGACACCGAGATGGAAGAAGTTGATCTTCGTACGCTGCAGGTCGA
Hsc82C3	CAAATTTATATAATATATAAAACATGAAGGCGAAAAAAGAGACACTATAGGGAGACCGGCAG
Hsc82Cdiafw	CAATTCTACTAAATCTGTCGATGAA
Hsp82C5	GTTGAAGAGGTTCCAGCTGACACCGAAATGGAAGAGGTAGATCTTCGTACGCTGCAGGTCGA
Hsp82C3	GTTATATTATGTTTTGTTTATAACCTATTCAAGGCCATGATGTTCTACCACTATAGGGAGACCGGCAG
Hsp82Cdiafw	CTCTACCAAGTCCGTAGATGAG
Kem1mc5	CAAAGCAATGCTGCTGACCGTGATAATAAAAAAGACGAATCTACTCGTACGCTGCAGGTCGAC
Kem1loxRev	GTAACCTCGAATATACTTCGTTTTTAGTCGTATGTTCTACATAGGCCACTAGTGGATCTG
Kem1Cdia	CAATATTCTGATGGTATAGGCAG
TagRFPT293rev	GTGACTCTTTCCCAAGTGAAACC
kanN	GCAGCGAGGAGCCGTAAT
Yef3mc5	GAAGGAATTGGGTGATGCTTACGTTTCTTCTGACGAAGAATTCCGTACGCTGCAGGTCGAC
Yef3mc3	CAATAAAAATCTTAAAGAAAACTGTGAAAGCAGTGATCAAAAAGAATCGATGAATTCGAGCTCG
yef3contr	TTTGGCTTCTGGTCAATTCC
Sup35mc5	CAAGGTACCACAATAGCAATTGGTAAAATTGTTAAAATTGCCGAGCGTACGCTGCAGGTCGA
Sup35C	GATCGGTATTATTGTGTTTGCATTTACTTATGTTTGCAAGAAATCATAGGCCACTAGTGGATCTG
Sup35Cdia	CCGGTCATATCAAAAAGGGTC
YGR054wC5	CTGAAGAAAAAGTGTTGAAAGATTTGGAAAAGTTGGGTTGGAAGGATGAACTTCGTACGCTGCAGGTCGA
YGR054wC3	GACGTTGTTAATATTTACACAGTTGTATGGATACATCAGTTTCTTCTAGTCACTATAGGGAGACCGGCAG
YGR054wCdiafw	CCTCACGCAAGAAGAACAGG
MMM1C5	GTATGTGGCCACGTAGTAAAAATACGAGAGAAGAAAAGCCTACAGAGTTACTTCGTACGCTGCAGGTCGA
MMM1C3	CACTTGGAGAATAGCCACTTGCACTAACCAAAAAATTTGTACATAAATATCACTATAGGGAGACCGGCAG
MMM1Cdiafw	CCCCATTTTCTCGAATTGCAG

MDM34C5	CTTCAAATAACTGGAAATGGGGCATGGAGGATAGCCCCCCACCATATCATCTTCGTACGCTGCAGGTCGA
MDM34C3	ATCGGAGAGTATGTATTTGTGTAGTTATGTACTTAGATATGTAACTTAATCACTATAGGGAGACCGGCAG
MDM34Cdiafw	GCCATTCTACTGTTCCCAC
Gem1C5	TATGTTCCTTTACGTTAATGAAATTATTCAAATCATCAAAATTCTCAAAACTTCGTACGCTGCAGGTCGA
Gem1C3	AGATATCATAGAAATGCAACACTTCCCTAATATAGAAATTTGGGCATTAACACTATAGGGAGACCGGCAG
Gem1Cdiafw	CTTGGAGAACAGGAATACGCC
