# Supplementary material for: eIF3a Destabilization and TDP-43 Alter Dynamics of Heat-Induced Stress Granules
Source: Int J Mol Sci. 2021 May 13;22(10):5164. doi: 10.3390/ijms22105164 (PMC8153170; doi:10.3390/ijms22105164)

Figure S1. Biochemical and microscopic analysis of WT Rpg1-GFP and mutant Rpg1-3-GFP under robust heat shock. (**A**) Polysome profile analysis of exponentially growing cells carrying Rpg1-3-GFP and Rpg1-GFP at physiological temperature of 25°C and upon robust heat shock at 46°C for 10 min. Arrows indicate positions of the 40S, 60S, monosome, and polysomes. Live-cell imaging of exponentially growing cells carrying Rpg1-3-GFP (**B**) or Rpg1-GFP (**C**) with another tagged eIF3 subunit - Nip1-RFP, Prt1-TagRFP-T, or Hcr1-TagRFP-T after HS for 10 min at 46°C. After deconvolution with the AMLE filter (Xcellence software, Olympus), single representative layers of Z-stacks are presented. Scale bars, 5μm.

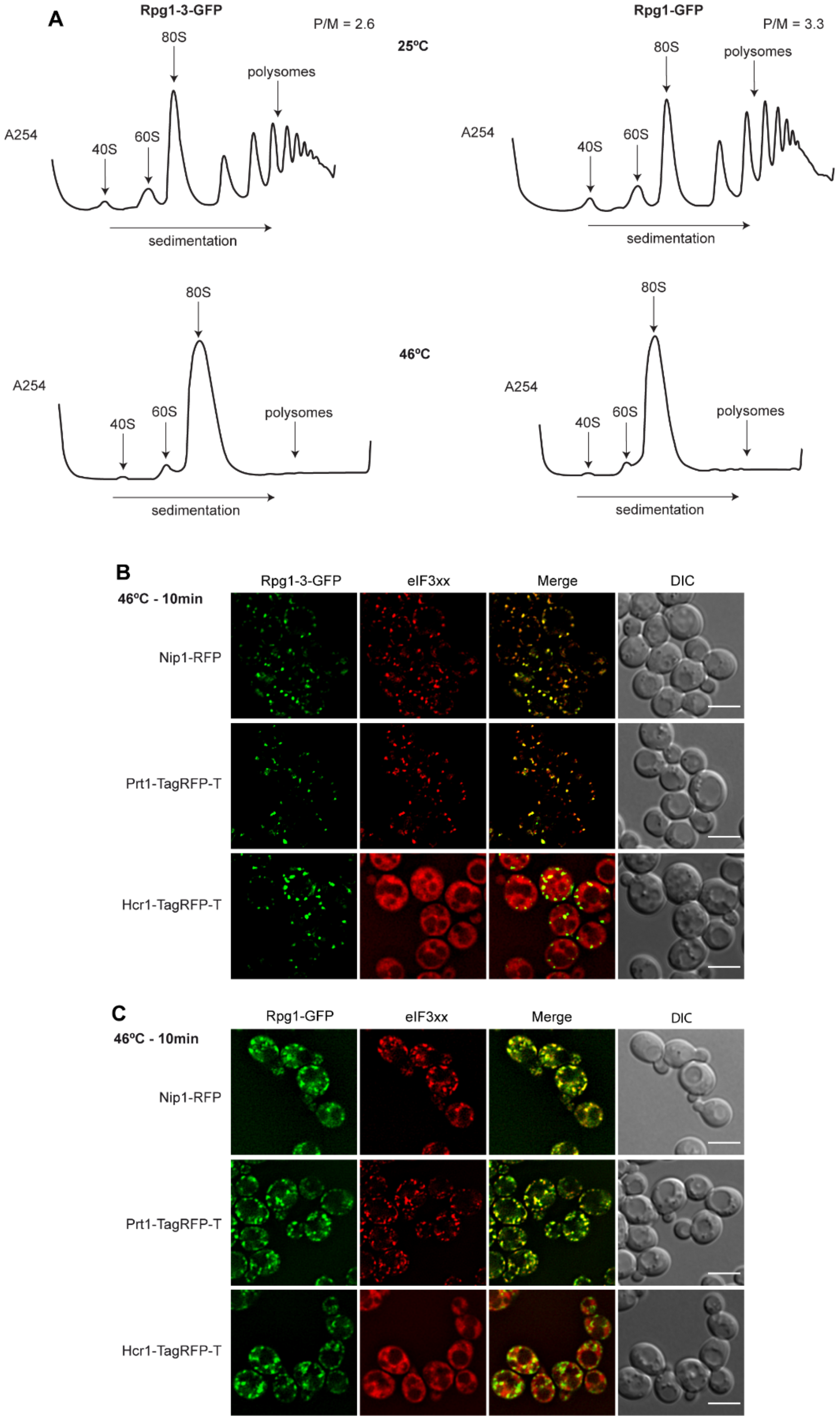

Supplement: Supplementary file 1 [file ijms-22-05164-s001.zip › Malcova et al Figure S1.pdf]
