# Supplementary material for: eIF3a Destabilization and TDP-43 Alter Dynamics of Heat-Induced Stress Granules
Source: Int J Mol Sci. 2021 May 13;22(10):5164. doi: 10.3390/ijms22105164 (PMC8153170; doi:10.3390/ijms22105164)

Figure S2. Cellular distribution of eIF3 subunits in *RPG1* and *rpg1-1* cells upon moderate heat shock. (**A**) Live-cell imaging of exponentially growing cells carrying the Rpg1-GFP and other eIF3 complex subunits upon heat shock for 30 min at 42°C. (**B**) Single-labeled Prt1-TagRFP-T strain after HS for 30 min at 42°C. (**C**) Live-cell imaging of exponentially growing cells carrying the Rpg1-1 variant and the eIF3 complex subunit after HS for 30 min at 42°C. Single representative layers of Z-stacks after deconvolution with the AMLE filter (Xcellence software, Olympus) are shown. Scale bars, 5μm. (**D**) 3D-brightness projections of a representative cell carrying Rpg1-1-GFP after HS at 42°C.

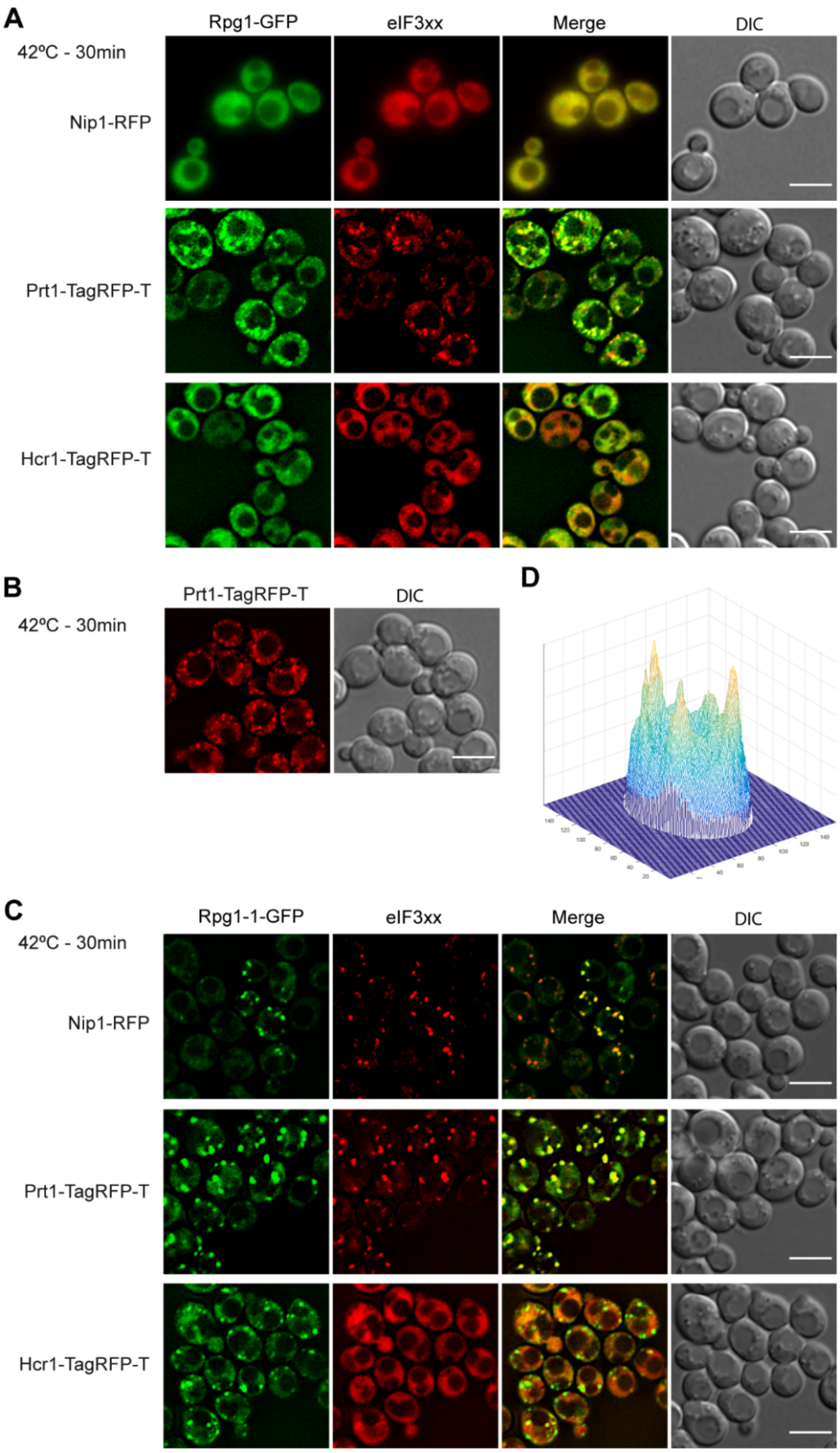

Supplement: Supplementary file 1 [file ijms-22-05164-s001.zip › Malcova et al Figure S2.pdf]
