# Supplementary material for: eIF3a Destabilization and TDP-43 Alter Dynamics of Heat-Induced Stress Granules
Source: Int J Mol Sci. 2021 May 13;22(10):5164. doi: 10.3390/ijms22105164 (PMC8153170; doi:10.3390/ijms22105164)

Figure S3. Cellular distribution of Yef3, Sup35, or eIF2A/YGR054w upon heat shock. Live-cell imaging of exponentially growing cells carrying Rpg1-GFP with endogenous TagRFP-T-fusions of either Yef3, Sup35, or eIF2A/YGR054w heat-shocked at 42°C for 30 min (**A**). Live-cell imaging of exponentially growing cells carrying Rpg1-3-GFP and Rpg1-GFP with endogenous TagRFP-T-fusion of eIF2A/YGR054w heat-shocked at 46°C for 10 min (**B**). Single representative layers of Z-stacks are presented. Scale bars, 5μm.

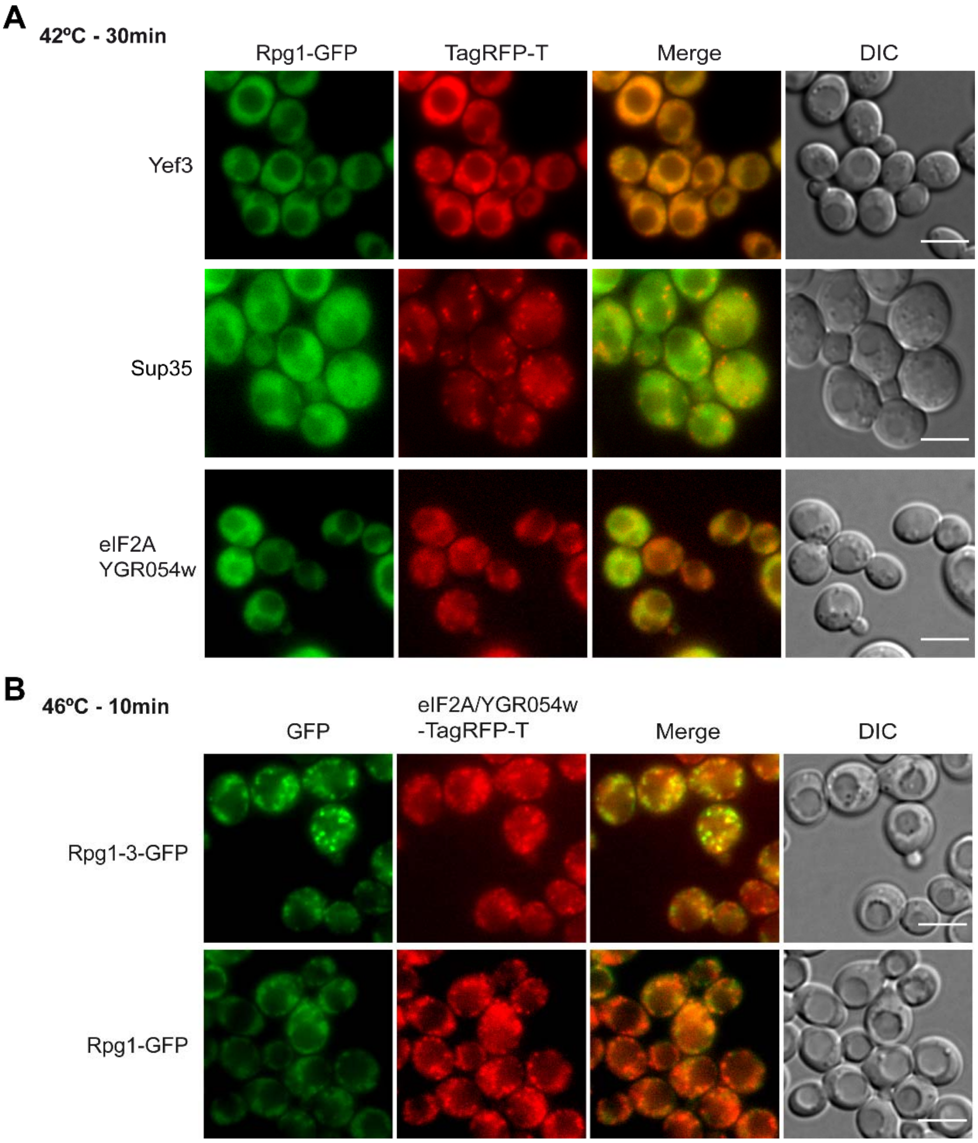

Supplement: Supplementary file 1 [file ijms-22-05164-s001.zip › Malcova et al Figure S3.pdf]
